# Changes in Children’s Dietary Intake of Selected Packaged Products Between 2019 and 2021 in the Context of Peru’s Front-of-Package Warning Label Policy: A Longitudinal Cohort Study

**DOI:** 10.3390/nu18213504

**Published:** 2026-10-25

**Authors:** Lorena Saavedra-Garcia, Kiomi Yabiku-Soto, Mayra Meza-Hernández, Caitlin M. Lowery, Donna R. Miles, Francisco Diez-Canseco, Lindsey Smith Taillie

**Affiliations:** 1 CRONICAS Center of Excellence in Chronic Diseases, Universidad Peruana Cayetano Heredia, Lima 15074, Peru; kiomi.yabiku@gmail.com (K.Y.-S.); mayrameza24@gmail.com (M.M.-H.); francisco.diez.canseco.m@upch.pe (F.D.-C.); 2 Department of Nutrition, Gillings School of Global Public Health, University of North Carolina, Chapel Hill, NC 27516, USA; cmlowery@wisc.edu (C.M.L.); taillie@unc.edu (L.S.T.); 3 Carolina Population Center, University of North Carolina, Chapel Hill, NC 27516, USA; drmiles@email.unc.edu

**Keywords:** food labeling, food policy, front-of-package labeling, ultra-processed foods, dietary intake

## Abstract

**Background/Objectives**: This study aimed to describe changes in children’s dietary intake of selected packaged food and beverage categories that commonly include products high in sugar, sodium, or saturated fat (HFSS packaged products) between 2019 and 2021, in the context of the implementation of Peru’s front-of-package warning label policy. **Methods**: A longitudinal panel analysis was conducted in four districts of Lima, Peru. Baseline data were collected face-to-face in 2019 and follow-up data by telephone in 2021. Intake was assessed using a semi-quantitative food frequency questionnaire and converted to daily amounts. Energy and nutrient estimates (total sugars, saturated fatty acids, sodium) were derived from product labels corresponding to each study period. Changes between 2019 and 2021 were tested using McNemar tests and quantile regression; analyses were run in Stata v.17. **Results**: Of 1283 mothers of children aged 3–5 years enrolled at baseline, 1032 completed follow-up (80.4%). Between 2019 and 2021 the prevalence of consumption decreased for some categories, including breakfast cereals and bars (78% to 63%, *p* < 0.001) and desserts (50% to 32%, *p* < 0.001), while it increased for others. Median daily energy intake per consumer from food and beverages decreased from 487.3 to 373.2 kcal (*p* < 0.001). Median total sugar intake decreased from 45.1 to 31.4 g/day, and sodium from 469.4 to 399.4 mg/day (both *p* < 0.001); saturated fatty acids showed a small decline (8.4 to 7.0 g/day, *p* < 0.001). **Conclusions**: Among participating children, estimated energy and nutrient intake from the selected HFSS packaged products declined overall, although changes varied across categories. These findings document temporal changes in the context of FOPWL implementation but cannot isolate the contribution of the policy from other concurrent factors.

## 1. Introduction

Non-communicable diseases (NCDs), such as obesity, type 2 diabetes, and cardiovascular diseases, continue to rise globally and have been closely associated with poor dietary patterns, particularly the increasing consumption of ultra-processed foods (UPF) [1,2]. UPFs are industrial formulations composed largely of refined ingredients, food additives, and preservatives, often lacking whole or minimally processed food [3]. These products are highly palatable and frequently ready-to-eat, making them convenient and attractive options whose availability and demand continue to grow [4]. According to the Pan American Health Organization (PAHO), sales of UPF grew by 26% across Latin America between 2000 and 2013 [5], with Peru exhibiting the highest increase in per capita sales, from 179 kcal per capita/day in 2009 to 207 kcal per capita/day in 2014 [5].

In high-income countries, UPFs contribute to more than half of total energy intake—as seen in the United States [6] and the United Kingdom [7]—and represent nearly 30% in countries like South Korea [8]. Although minimally processed and processed foods still represent a significant component of dietary patterns in many Latin American countries, the consumption of UPFs is rapidly increasing [9,10]. For example, in Colombia, the proportion of total energy intake from UPFs increased from 15.9% in 2005 to 19.2% in 2015 [11]. Meanwhile, Brazil and Chile report contributions of 19.7% in 2017–2018 [12] and 28.6% in 2010 [13], respectively. These differences suggest that Latin American countries are undergoing distinct stages of nutritional transition, reflecting variations in dietary patterns that may be influenced by diverse socioeconomic dynamics [14]. In response, since Ecuador first implemented a front-of-package labeling policy in 2014 and Chile introduced the first octagonal warning label system in 2016, several Latin American countries have adopted similar national policies [15].

In Peru, Law 30021: Promotion of Healthy Eating for Children and Adolescents established the use of front-of-package warning labels (FOPWLs) to inform consumers about products exceeding thresholds for total sugar, saturated fat, sodium, and trans fats [16]. The FOPWLs consist of black octagonal warnings with white text displayed on the front of the package (Figure 1). After a lengthy policy process, black octagon warnings were implemented through a phased approach. The first phase was implemented in June 2019, followed by a second phase with stricter nutrient thresholds implemented in September 2021. Previous work has documented the design and approval process of the Peruvian FOPWL policy [17], as well as consumers’ awareness and perceptions about FOPWL; however, evidence on changes in dietary intake, particularly from longitudinal studies, remains limited. Given that the law was designed to protect children—who are particularly vulnerable to the marketing and availability of unhealthy packaged foods—monitoring dietary intake during these implementation phases is essential to document changes over time, support future evaluations of the policy, and inform preventive strategies aimed at reducing the burden of non-communicable diseases.

Warning labels in Spanish, as displayed on products marketed in Peru, are shown on the left; English translations are shown on the right.

The objective of this study was to describe changes in children’s dietary intake of selected packaged food and beverage categories that commonly include products high in sugar, sodium, or saturated fat (hereafter, HFSS packaged products) between 2019 and 2021, in the context of the implementation of FOPWL. Data included a longitudinal cohort of children aged 3 to 5 years in 2019, with follow-up data collected in 2021 when the children were approximately 5 to 8 years old.

## 2. Materials and Methods

### 2.1. Study Design

This study is a longitudinal analysis based on data from a panel study designed to assess changes in food consumption and nutrition-related practices among children in the context of the implementation of mandatory FOPWLs.

### 2.2. Setting and Sampling Scheme

The study sample was drawn from households with children aged 3 to 5 years living in four urban districts of Lima, the capital of Peru: Los Olivos and San Martín de Porres in the northern area, and San Juan de Miraflores and Santiago de Surco in the southern area. Based on household per capita income at the census-block level, these urban districts represent a broad range of socioeconomic levels (SEL), from low-income to middle-class households.

A stratified sampling method was employed to ensure representation across the socioeconomic strata included in the study and across the four selected districts. Sample size calculations were conducted to guarantee adequate statistical power for detecting changes in food and beverage consumption over time. Additional details on the sampling procedures and power calculations are provided in the Appendix A.

### 2.3. Participants

Baseline data was collected in April–June 2019. Eligible participants were women aged 18 years or older, living in one of the four selected districts, and serving as the primary caregivers responsible for feeding children aged 3 to 5 years. Mothers were excluded if they had a visual, auditory, or speech impairment that could interfere with survey participation, or if their child had chronic health conditions requiring a medically prescribed diet. Additionally, children who had undergone temporary dietary modifications due to illness in the 30 days prior to data collection were not eligible. At baseline in 2019, a total of 1283 mothers participated in the study.

The same cohort was reassessed in May–September 2021, when the children were approximately 5 to 8 years old. A total of 80.4% (*n* = 1032) participated in the follow-up survey; 251 participants (19.6%) were excluded from analysis because they were unable to be located or declined participation in the follow-up survey (Figure 2). Baseline sociodemographic characteristics of participants retained in the study and those lost to follow-up were compared to assess potential differences between groups (Supplementary Table S1).

### 2.4. Procedures

Baseline data were collected in 2019 through face-to-face home interviews conducted by trained dietitians. Prior to data collection, a household mapping and screening process was conducted, during which fieldworkers visited households door to door, introduced the study, and assessed eligibility. Once eligibility was confirmed, mothers provided written informed consent and completed a structured questionnaire administered by the interviewer. The questionnaire included several modules, covering sociodemographic and household characteristics, children’s dietary intake assessed through a food frequency questionnaire (FFQ), and items related to nutrition-related behaviors and perceptions of the implementation of front-of-package warning labels.

Due to COVID-19 restrictions, follow-up data were collected via telephone interviews conducted by the same team. The overall structure and sequence of the questionnaire modules were maintained, and in both rounds, mothers completed the same FFQ.

In both years, interviews lasted approximately 20 to 25 min. Responses were recorded in REDCap, a secure web application hosted by Universidad Peruana Cayetano Heredia. All interviews were recorded, and 30% of them were randomly reviewed by supervisors within 24 h to detect and correct inconsistencies or potential errors. Participants were recontacted if necessary to confirm or clarify responses.

### 2.5. Dietary Assessment

#### 2.5.1. Food Frequency Questionnaire

A semi-quantitative food frequency questionnaire (FFQ) was developed to assess the intake of foods and beverages among preschool-aged children living in urban areas of Lima. To develop the FFQ food list, item selection was informed by three 24-h dietary records completed by a sample of 42 mothers of children aged 3 to 5 years, who live in La Molina and San Juan de Lurigancho districts located in the eastern part of Lima. Diet recalls consisted of maternal report of all foods and beverages the child had consumed during the previous 24 h specifying portion sizes and product brands. Common items were included in the FFQ for the current study to ensure cultural and contextual relevance to the target population.

The FFQ consisted of 14 beverage and 17 food categories (see Supplementary Material S1. Food Frequency Questionnaire). The present analyses focused on selected packaged food and beverage categories, including categories that commonly contain products high in sugar, sodium, or saturated fat (HFSS). Water and plain milk were retained to characterize beverage intake more comprehensively; thus, the HFSS designation reflects the predominant analytical focus and category-selection rationale and is not intended as a nutrient-profile classification of every category or individual product included in the analyses.

The present analyses included 7 beverages and 11 food categories; homemade beverages such as fresh juices and traditional drinks were excluded and categories with similar product compositions were consolidated for the current study. The categories for analyses were:

- Beverages (*n* = 7): Water, nectars and flavored fruit drinks (e.g., packaged fruit drinks, powdered drink mixes), carbonated and sports drinks (soda, sports drinks), teas, milk (e.g., evaporated whole milk, low-fat milk), dairy drinks (e.g., flavored milk-based beverages, drinkable yogurts), and formulas and supplements.
- Foods (*n* = 11): salty crackers, sweet crackers (e.g., cookies, including filled and chocolate-coated varieties), sweet pastries and bakery products (e.g., cakes and pastries), breakfast cereals and bars, candy and chewables (e.g., candies, gummies, and marshmallows), desserts (e.g., gelatin desserts), chocolate candies (e.g., chocolate confectionery and chocolate-coated candies), instant soups, processed meats (e.g., hot dogs, cold cuts, nuggets), dairy desserts (e.g., ice cream and yogurt with cereal), and snacks (e.g., potato and corn-based chips).

Participants were asked to report the child’s consumption during the previous 30 days. The FFQ response format was open-ended, allowing participants to report intake in terms of daily, weekly, or monthly frequency. For beverages, six portion size options were provided; for foods, a standard portion size was assigned based on the most reported values from the dietary recall study.

To support portion size estimation, standardized images of food and beverage portions were used during both data collection rounds. In 2019, images were shown to participants during the in-person interview. In 2021, participants were sent a link to view the same standardized images online during the telephone-based interview. These images were displayed in real time by the interviewer using the Slides application, allowing participants to visually reference portion sizes while completing the FFQ (see Supplementary Material S1).

The FFQ was subsequently evaluated in an independent sample of 118 mothers against three non-consecutive 24-h dietary recalls (two weekdays and one weekend day administered one week apart. Mean daily nutrient intake across the three recalls was calculated and compared with FFQ estimates for the corresponding food and beverage categories.

#### 2.5.2. Food Composition Tables

Reported intake frequencies were converted to daily consumption estimates, from which volume (mL/day) and weight (g/day) were calculated for beverages and foods, respectively. Energy (kcal), total sugars (g), saturated fatty acids (g), and sodium (mg) intake were estimated for each item using a food composition table developed from the nutritional information available on product labels, which were photographed in supermarkets throughout Metropolitan Lima [18]. Data collected in 2019 were used to estimate intake for the first study wave, while photographs collected in 2020 and 2021 were used for the second wave. Photographed products were grouped according to the same food and beverage categories used in the FFQ, and for each category the mean nutrient values were calculated by averaging the declared content per 100 g or 100 mL across all products.

### 2.6. Sociodemographic Variables

Demographic characteristics of mothers included: age, marital status (married, cohabiting, single, separated/divorced, or widowed), number of children, educational attainment [primary education (0–6 years), secondary education (7–11 years), and higher education (≥12 years)], and maternal occupation (working or homemaker). Household socioeconomic level (SEL) was classified according to the “Stratified Plans of Metropolitan Lima at the Block Level by Household Per Capita Income,” developed by the National Institute of Statistics and Informatics [19].

For children, sex (male/female) was recorded, and age was calculated based on the difference between the interview date and the child’s date of birth.

### 2.7. Statistical Analysis

Descriptive statistics were used to characterize the consumption of HFSS packaged products reported in the study. The analysis included the frequency of consumption, mean daily intake (in grams for foods and milliliters for beverages), and the corresponding energy and nutrient contributions. For each food and beverage category, the median, and quartiles (Q1–Q3) were calculated. Energy intake (kcal), total sugars (g), saturated fatty acids (g), and sodium (mg) were estimated per category based on the FFQ. To minimize the influence of extreme values and potential reporting errors, observations exceeding the 99th percentile calculated separately for each food and beverage category and survey wave were excluded from the analysis. Exclusions were applied independently by wave; therefore, excluding an observation in one wave did not entail excluding the corresponding observation for the same child in the other wave.

To examine differences in consumption between 2019 and 2021, paired and quantile regression analyses were conducted. First, the McNemar test was applied to assess differences in the proportion of participants reporting consumption of each food and beverage category between baseline and follow-up. Second, quantile regression was used to estimate differences between 2019 and 2021 in median daily intake (mL or g), energy intake (kcal), and nutrient intake—total sugars and saturated fatty acids (g) and sodium (mg)—from HFSS packaged products among participants who reported consumption at both time points [20]. Quantile regression models were fitted using the Stata qreg2 command, with survey wave included as the independent variable and standard errors clustered by child identifier to account for intra-child correlation. Wave-specific estimates and between-wave contrasts were obtained using margins year and margins r.year, respectively. Confidence intervals and *p*-values for the contrasts were based on Wald inference. The wave coefficient estimates the difference between wave-specific medians among the retained observations and does not represent the median of individual within-child changes. Statistical significance was set at *p* < 0.05. All analyses were conducted using Stata version 17 (StataCorp, College Station, TX, USA).

### 2.8. Ethics Statement

The Institutional Review Boards (IRB) of the University of North Carolina at Chapel Hill (United States) and Universidad Peruana Cayetano Heredia (Peru) approved the study protocol (SIDISI 103427). All participants provided written informed consent at baseline, agreeing to participate in the follow-up assessments. As recognition for their time, participants received crayons and coloring books for their children in 2019, and a supermarket gift card valued at approximately 10 USD in 2021.

## 3. Results

Table 1 includes the sociodemographic characteristics of the baseline sample. Most mothers had higher education (68.4%; *n* = 706), were cohabiting (46.7%; *n* = 482), and were homemakers (73.2%; *n* = 755). The sample included similar proportions of households in socioeconomic levels B (49.5%; *n* = 511) and D (50.5%; *n* = 521). Children had a mean age of 4.0 years (SD 0.8) at baseline, and the distribution by sex was 46.1% female (*n* = 476) and 53.9% male (*n* = 556). Compared with participants retained in the study, mothers lost to follow-up had lower educational attainment (*p* < 0.001); no statistically significant differences were observed for the other sociodemographic characteristics assessed (Supplementary Table S1). Retention differed by maternal education: 44.4% (16/36) of mothers with primary education, 76.2% (310/407) with secondary education, and 84.0% (706/840) with higher education were retained (*p* < 0.001).

### 3.1. Consumption of HFSS Packaged Products

In the independent evaluation of the FFQ, correlations with the 24-h dietary recalls varied across food and beverage categories and were generally low to moderate. Correlations were higher for carbonated and sports drinks, formulas and supplements, and snacks; adjustment for socioeconomic level, age, and sex resulted in minimal changes to these estimates (Supplementary Tables S2 and S3).

Table 2 shows the prevalence of reported consumption for each food and beverage category. The proportion reporting milk consumption increased from 55.5% in 2019 to 61.1% in 2021 (*p* = 0.003). Declines in consumption were observed for breakfast cereals and bars (78.4% to 62.9%, *p* < 0.001) and desserts (49.7% to 31.8%, *p* < 0.001). Smaller decreases were observed for dairy drinks (90.9% to 88.1%, *p* = 0.019), formulas and supplements (16.3% to 12.6%, *p* = 0.006), salty crackers (67.6% to 61.0%, *p* < 0.001), and candy and chewables (67.6% to 62.1%, *p* = 0.004). Consumption of chocolate candies increased from 58.7% to 65.1% (*p* < 0.001), as did instant soups (15.2% to 19.2%, *p* = 0.004) and processed meats (76.9% to 80.7%, *p* = 0.018).

### 3.2. Daily Intake by Volume

Table 3 reports median daily food and beverage intake among children who consumed the corresponding category in both 2019 and 2021. Overall beverage intake remained stable over time (1425.7 mL vs. 1432.6 mL, *p* = 0.850). However, consumption of water increased (718.2 mL to 900.0 mL, *p* < 0.001) while decreases were observed for dairy drinks (157.5 mL to 126.0 mL, *p* < 0.001). Overall total food intake decreased over time with the largest reduction found among dairy desserts (17.8 g to 10.6 g, *p* < 0.001). However, instant soups showed a significant increase (24.0 g to 36.0 g, *p* = 0.030). When stratified by socioeconomic level, the direction and magnitude of changes in consumption were generally consistent across groups, with minor differences observed in selected food categories (Supplementary Table S4).

### 3.3. Daily Energy Intake

Among consumers, quantile regression showed reductions in energy intake from HFSS packaged products between 2019 and 2021 (Table 4). The median daily caloric intake per consumer from assessed HFSS packaged products decreased from 487.3 kcal in 2019 to 373.2 kcal in 2021 (*p* < 0.001). Declines were significant for dairy drinks (−20.8 kcal, *p* < 0.001), dairy desserts (−9.9 kcal, *p* < 0.001) and nectars and flavored fruit drinks (−1.4 kcal, *p* < 0.001). In contrast, caloric intake increased among sweet pastries and bakery products (+10.3 kcal, *p* < 0.001), instant soups (+7.5 kcal, *p* = 0.027), processed meats (+3.9 kcal, *p* = 0.022), and chocolate candies (+1.7 kcal, *p* = 0.028).

### 3.4. Daily Intake of Total Sugar, Sodium and Saturated Fatty Acids

Median consumption intakes are reported for total sugar, sodium and saturated fatty acids (Table 5). Median sugar consumption decreased from 45.1 g/day in 2019 to 31.4 g/day in 2021 (*p* < 0.001). Sodium intake also declined modestly (469.4 mg/day vs. 399.4 mg/day, *p* < 0.001). The overall median intake of saturated fatty acids remained relatively stable, from 8.4 g to 7.0 g (*p* < 0.001). Decreases in sodium intake were significant among dairy drinks and dairy desserts, while increases in sodium were observed among instant soups and processed meats.

## 4. Discussion

This study documents changes in children’s reported consumption of selected HFSS packaged products between 2019 and 2021, the period in which the first phase of Peru’s FOPWL policy was implemented. The overall estimated intake of energy, total sugar, sodium, and saturated fatty acids from the assessed packaged products declined between study waves among participants who consumed the corresponding categories at both waves. However, these changes were not uniform: while declines in products such as dairy desserts and dairy drinks are encouraging, increases in items like instant soups, chocolate candies, and processed meats suggest that some product categories commonly regarded as less healthful continued to contribute to children’s dietary intake.

Between 2019 and 2021, median intake from the assessed HFSS packaged products declined by 13.6 g/day for total sugars and 70 mg/day for sodium, while saturated fatty acids showed a smaller reduction. The decline in total sugars may partly correspond to lower free sugar intake, particularly from beverage categories containing added sugars, and may therefore represent a favorable change toward the World Health Organization’s conditional recommendation that free sugars account for less than 5% of daily energy intake [21]. Similarly, the reduction in sodium from these products may contribute to lowering children’s overall intake in line with WHO recommendations [22]. However, these estimates cannot be directly compared with the recommendations because the study measured total sugars and sodium only from selected HFSS packaged products, excluding other dietary sources—including homemade sweetened beverages and discretionary salt—and did not assess total energy intake. The observed changes may reflect both reduced consumption of certain categories and product reformulation, including the increased use of non-nutritive sweeteners documented after FOPWL implementation [18,23].

Evidence from a Chilean cohort study shows reductions in children’s intake of total sugars, saturated fatty acids, and sodium at school after the food labeling, marketing, and school sales regulations took effect, although some compensatory increases were observed outside the school setting [24]. The reduction in nutrients of concern observed in our study is directionally consistent with these findings; however, the studies’ designs differ, and results should therefore be compared with caution. In addition, the policies differed: Chile implemented warning labels alongside comprehensive restrictions on child-directed marketing, whereas Peru’s accompanying measures were more limited in scope and followed a different implementation schedule.

In the present study, although the proportion of consumers of some HFSS packaged products declined between survey years, certain categories—such as instant soups, chocolate candies and processed meats—showed increases in the number of consumers, suggesting potential replacement effects. Evidence from Chilean household purchases showed that reductions in purchases of products classified as high in nutrients of concern were accompanied by increases in not-high-in products within the same food categories, potentially reflecting consumer choice, product reformulation, or both [25]. Because our analyses did not assess within-child substitution across categories or include foods prepared at home, we cannot determine whether replacement occurred among packaged product categories or between packaged and home-prepared foods. Complementing this quantitative evidence, a qualitative study with Peruvian mothers of preschoolers documented self-reported reductions in certain purchases but lower responsiveness to warnings for products considered essential or already perceived as unhealthy [26]. Because our analyses did not assess within-child substitution, foods prepared at home, or mothers’ perceptions and use of warning labels, we cannot determine whether the category-specific changes reflected product replacement, differential responsiveness to warnings, or both. Assessing the overall diet together with label-related perceptions and purchasing practices is therefore necessary to evaluate these potential mechanisms.

Although this study was designed to examine changes in dietary intake in the context of Peru’s FOPWL implementation, other contextual factors should be considered. Baseline data were collected from April to June 2019, with recruitment occurring shortly before the first implementation phase in June; therefore, the assessment may not represent a period entirely before implementation because products bearing warning labels may already have been available. Children were also approximately two years older at follow-up, and age-related changes in energy requirements, food preferences, and dietary autonomy may have influenced intake independently of the policy. Seasonality may also have contributed, as baseline data were collected in autumn/winter and follow-up extended from autumn/winter into early spring [27]. National data showed a higher energy contribution from ultra-processed foods among Peruvian children during April–June than during January–March, whereas estimates for July–September did not differ significantly from January–March [28]. However, the product classifications were not directly comparable with those used in our study, and because each data-collection period corresponded to a specific study wave, seasonal effects cannot be separated from other temporal changes.

Because the data-collection period was linked to each study wave, the present design cannot separate seasonal effects from other changes occurring between 2019 and 2021. Beyond potential seasonal effects, increases in some product categories may reflect other mechanisms, including consumer preferences and continued market availability. For example, chocolate confectionery represents a highly palatable, indulgent food, consistent with evidence showing a stronger preference for sweet tastes among children than adults [29]. However, these mechanisms were not directly assessed in our study.

The COVID-19 pandemic may also have contributed to dietary changes through school closures, mobility restrictions, altered household routines, and economic constraints [30]. In a study among infants and young children from low-income urban areas of Peru, 90.9% of respondents reported lower household income and, among households that adjusted their expenditures, 61.3% reduced food spending. Sweet food consumption declined by 15.0 percentage points, from 33.1% before the pandemic to 18.1% during the pandemic, whereas sugar-sweetened beverage consumption remained stable [31]. This reduction was broadly similar to those observed for breakfast cereals and bars and dairy desserts in our study, although differences in age, food definitions, and assessment methods limit direct comparison. Reduced food expenditure was proposed as one possible explanation for these changes [31]. Nutritional education during remote schooling may also have contributed, as Peru’s “Aprendo en casa” initiative included health and nutrition content [32]. These contextual factors support the pandemic as a plausible contributor but do not establish it as the primary driver or distinguish its contribution from that of FOPWLs and other concurrent measures.

This study provides one of the few longitudinal assessments of changes in food consumption among Peruvian children, the priority population under Peru’s Law 30021. Although the study design cannot isolate the specific contribution of FOPWL policy, the findings provide decision-makers with evidence on how consumption evolved within the broader context of healthy eating policies and concurrent societal changes. Future studies should include other age groups and populations from different regions of Peru, and incorporate longer-term follow-up with repeated measurements to determine whether dietary changes are sustained, extend beyond packaged products, or evolve over successive years of policy implementation.

This study has some limitations. First, dietary intake was reported by mothers using an FFQ, which may be subject to recall and social desirability bias. Additionally, the FFQ showed low-to-moderate correlations with the three non-consecutive 24-h dietary recalls, with variability across food and beverage categories, suggesting that estimates for categories with lower correlations may be subject to greater measurement variability. Therefore, small category-specific changes should be interpreted cautiously. Because the comparison method consisted of modified 24-h dietary recalls focused on selected HFSS packaged products, the extent to which the observed low correlations reflect measurement error in the FFQ, the recalls, or both methods cannot be determined. However, the same FFQ was applied at both study waves, supporting consistency in the assessment of dietary intake over time. Under this consistent measurement approach, any systematic error inherent to the FFQ that remained stable over time would be expected to affect both waves similarly. In addition, nutrient intake was estimated using average values for food categories rather than product-specific information. This approach may not capture variability among products within each category or fully reflect changes associated with reformulation. However, using product-label information collected during the corresponding study periods improves the temporal relevance of the estimates. Second, data collection methods differed across waves—face-to-face in 2019 and telephone interviews in 2021—although standardized images were used in both to minimize errors. Telephone interviews limited the interviewer’s ability to observe non-verbal cues that could indicate difficulties in understanding or responding to the FFQ; thus, differences in the interview setting may have affected the reporting process, although the direction or magnitude of this potential effect cannot be determined. Third, the follow-up took place during the COVID-19 pandemic, when children’s confinement at home may have influenced dietary intake. Fourth, the study focused exclusively on some HFSS packaged products, limiting interpretation in relation to total energy intake and overall dietary intake. Fifth, loss to follow-up differed by maternal education, with mothers with primary education less likely to remain in the cohort. This may have introduced selection bias if mothers who remained in the cohort were more health-conscious or more responsive to nutrition-related information, potentially overestimating favorable dietary changes among children. Finally, given its observational design, causal inferences about the impact of the policy cannot be made.

## 5. Conclusions

In conclusion, this pre–post study provides longitudinal evidence on changes in children’s consumption of selected HFSS packaged products in Lima between 2019 and 2021 in the context of Peru’s FOPWL implementation. Modest reductions in energy, sugar, sodium, and saturated fatty acids intake were observed as well as reductions in consumption of a number of packaged food and beverage groups, although these changes cannot be attributed to specific regulatory measures given the study design and the broader contextual changes occurring during this period. However, persistent high intake and increases in certain product categories indicate that changes were not uniform across the assessed food categories. Categories in which consumption persisted or increased should be prioritized for surveillance and further research to determine the roles of product substitution, availability, price, consumer preferences, and responsiveness to FOPWLs before defining complementary interventions. Future evaluations should assess the overall diet, include other age groups, and incorporate longer-term follow-up with repeated measurements to determine whether dietary changes are sustained, extend beyond the packaged product categories assessed, or evolve over successive years of policy implementation.

## Figures and Tables

**Figure 1:**
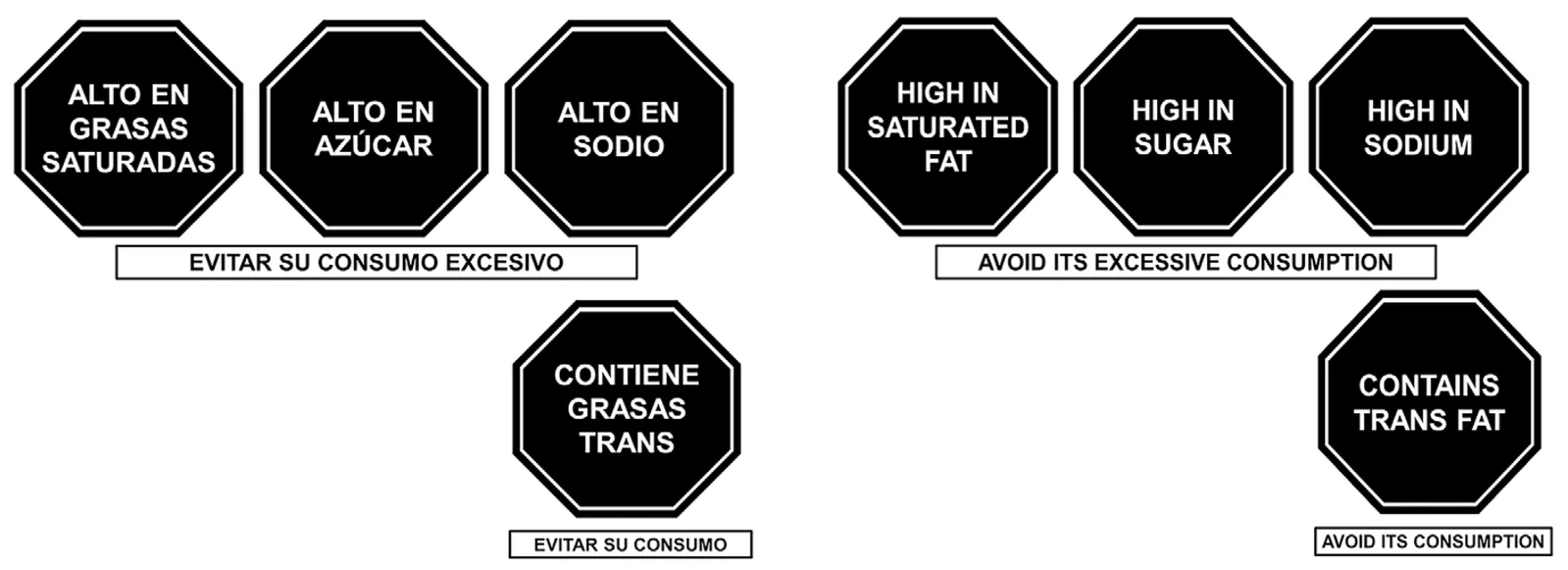
Peruvian front-of-package warning labels. Original warning labels in Spanish are shown on the left, with their corresponding English translations on the right.

**Figure 2:**
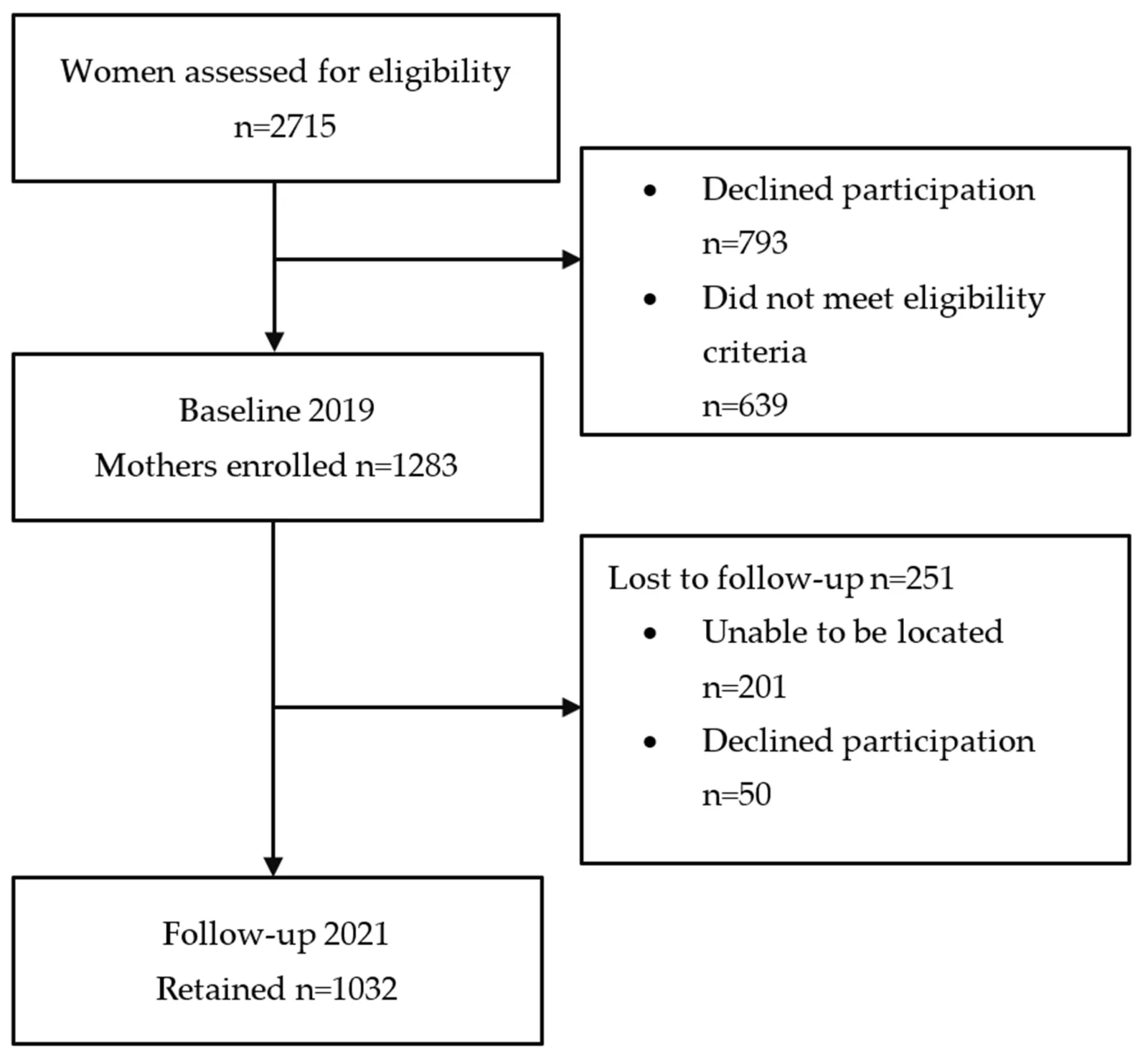
Sample size flowchart of participants recruited in 2019 and reassessed in 2021.

**Table 1:** Baseline sociodemographic characteristics of the mothers and children (*n* = 1032).

Characteristics	*n* (%)
Mother characteristics	
Age *	36.3 (7.2)
Education	
Primary (0–6 years)	16 (1.6)
Secondary (7–11 years)	310 (30.0)
Higher education (≥12 years)	706 (68.4)
Marital status	
Married	329 (31.9)
Cohabiting	482 (46.7)
Single	163 (15.8)
Separated/Divorced	55 (5.3)
Widowed	3 (0.3)
Socioeconomic level	
B	511 (49.5)
D	521 (50.5)
Main occupation	
Working	277 (26.8)
Homemaker	755 (73.2)
Number of children *	2.2 (1.1)
Children characteristics	
Age at baseline *	4.0 (0.8)
Sex	
Female	476 (46.1)
Male	556 (53.9)

* values are presented as mean (standard deviation).

**Table 2:** Number and percent of participants reporting consumption for each beverage and food group in 2019 and 2021.

Beverage/Food Category	2019		2021		Difference (%)	*p*-Value *
	n	%	n	%		
Total food and beverages	1032	100.0	1032	100.0	0.0	
Total beverages	1032	100.0	1031	99.9	−0.1	.
Water	1005	97.4	1003	97.2	−0.2	0.450
Nectars and flavored fruit drinks	673	65.2	630	61.1	−4.2	0.020
Carbonated and sports drinks	691	67.0	716	69.4	2.4	0.112
Teas	873	84.6	899	87.1	2.5	0.086
Milk	573	55.5	631	61.1	5.6	0.003
Dairy drinks	938	90.9	909	88.1	−2.8	0.019
Formulas and supplements	168	16.3	130	12.6	−3.7	0.006
Total food	1030	99.8	1028	99.6	−0.2	0.317
Salty crackers	698	67.6	629	61.0	−6.7	<0.001
Sweet crackers	854	82.8	848	82.2	−0.6	0.740
Sweet pastries and bakery products	494	47.9	474	45.9	−1.9	0.253
Breakfast cereals and bars	809	78.4	649	62.9	−15.5	<0.001
Candy and chewables	698	67.6	641	62.1	−5.5	0.004
Desserts	513	49.7	328	31.8	−17.9	<0.001
Chocolate candies	606	58.7	672	65.1	6.4	<0.001
Instant soups	157	15.2	198	19.2	4.0	0.004
Processed meats	794	76.9	833	80.7	3.8	0.018
Dairy desserts	787	76.3	674	65.3	−11.0	<0.001
Snacks	654	63.4	673	65.2	1.8	0.185

* *p*-value obtained from McNemar test assessing differences in the proportion of participants reporting consumption between 2019 and 2021.

**Table 3:** Per consumer daily intake (volume in g/ml) of HFSS packaged products in 2019 and 2021.

Beverage/Food Category	Consumers (n)	2019 Median (Q1–Q3)	2021 Median (Q1–Q3)	Median Difference 2021–2019 (95% CI)	*p*-Value *
Total beverages **^†^**	1030	1425.7 (986.3–1974.1)	1432.6 (1026.5–2009.3)	6.9 (−64.2, 78.0)	0.850
Water	978	718.2 (450–1159.4)	900.0 (505.6–1350)	181.8 (126.2, 237.3)	<0.001
Nectars and flavored fruit drinks	475	32.9 (14.1–77.1)	31.7 (14.1–65.8)	−1.2 (−6.3, 3.9)	0.651
Carbonated and sports drinks	572	27.0 (13.5–54)	30.0 (13.5–63)	3.0 (−0.2, 6.2)	0.066
Teas	792	94.5 (47.3–225)	98.7 (63–225)	4.2 (−10.4, 18.8)	0.573
Milk	420	225.0 (94.5–450)	225.0 (94.5–450)	0.0 (−49.0, 49.0)	1.000
Dairy drinks	841	157.5 (70–450)	126.0 (57.4–256.5)	−31.5 (−48.0, −15.0)	<0.001
Formulas and supplements	50	225.0 (100.8–470)	225.0 (94.5–450)	0.0 (−103.9, 103.9)	1.000
Total food	1026	56.9 (29.3–92.6)	44.0 (23.6–75.5)	−12.8 (−16.6, −9.0)	<0.001
Salty crackers	488	4.4 (1.9–8.8)	4.4 (1.9–8.8)	0.0 (−0.5, 0.5)	1.000
Sweet crackers	729	4.9 (3.1–10.5)	4.9 (3.1–9.8)	0.0 (−0.6, 0.6)	1.000
Sweet pastries and bakery products	276	4.0 (1.7–8)	6.8 (3.4–10.3)	2.8 (1.8, 3.9)	<0.001
Breakfast cereals and bars	552	5.6 (2.4–9.6)	5.4 (2.4–9)	−0.2 (−1.0, 0.6)	0.640
Candy and chewables	475	1.8 (0.9–3.6)	1.7 (0.8–3.5)	−0.1 (−0.3, 0.2)	0.540
Desserts	187	24.1 (10.3–64.4)	13.8 (6.9–32.2)	−10.4 (−22.8, 2.1)	0.104
Chocolate candies	443	1.9 (0.9–4.3)	2.2 (0.9–4.3)	0.3 (0.0, 0.6)	0.028
Instant soups	72	24.0 (12–56)	36.0 (12–56)	12.0 (1.3, 22.6)	0.030
Processed meats	681	6.6 (2.8–13.2)	8.0 (3.8–13.3)	1.4 (0.2, 2.6)	0.020
Dairy desserts	543	17.8 (7.6–38.1)	10.6 (4.1–20.4)	−7.3 (−9.2, −5.3)	<0.001
Snacks	480	1.2 (0.5–2.5)	1.6 (0.5–2.5)	0.3 (0.0, 0.7)	0.083

Note: values only include participants who were consumers in both 2019 and 2021; therefore, the number of participants varies across categories. * *p*-value obtained from quantile regression comparing median daily intake between 2019 and 2021. ^†^ Total beverages include water. CI = confidence interval.

**Table 4:** Per consumer daily energy (kcal) intake of HFSS packaged products in 2019 and 2021.

Beverage/Food Category	Consumers (n)	2019 Median (Q1–Q3)	2021 Median (Q1–Q3)	Median Difference 2021–2019 (95% CI)	*p*-Value *
Total food and beverages	1031	487.3 (309.6–713.3)	373.2 (233.5–546)	−114.1 (−137.6, −90.6)	<0.001
Total beverages	1030	298.2 (140.6–464.9)	197.3 (107.1–340.7)	−100.9 (−122.3, −79.5)	<0.001
Water	978	N.D.	N.D.	N.D.	N.D.
Nectars and flavored fruit drinks	475	7.8 (3.4–15.7)	6.4 (2.6–11.8)	−1.4 (−2.5, −0.3)	0.010
Carbonated and sports drinks	572	6.6 (3.3–13)	6.8 (3.3–15.3)	0.2 (−0.5, 0.9)	0.542
Teas	792	1.6 (0.8–3.8)	1.6 (1.1–3.8)	0.1 (−0.2, 0.3)	0.573
Milk	420	141.9 (59.6–283.8)	141.9 (59.6–283.8)	0.0 (−30.9, 30.9)	1.000
Dairy drinks	841	104.6 (46.3–302.3)	83.9 (38–169.5)	−20.8 (−31.7, −9.8)	<0.001
Formulas and supplements	50	147.8 (66.2–308.7)	147.8 (62.1–295.6)	0.0 (−68.2, 68.2)	1.000
Total food	1026	168.1 (94.5–261.3)	137.8 (76.2–228.2)	−30.3 (−41.2,−19.4)	<0.001
Salty crackers	488	19.5 (8.4–39)	19.5 (8.4–39)	0.0 (−2.2, 2.2)	1.000
Sweet crackers	729	23.4 (15.1–50.2)	23.4 (15.1–46.8)	0.0 (−2.8, 2.8)	1.000
Sweet pastries and bakery products	276	14.5 (6.2–29)	24.8 (12.4–37.3)	10.3 (6.5, 14.2)	<0.001
Breakfast cereals and bars	552	22.0 (9.4–37.7)	21.2 (9.4–35.3)	−0.8 (−4.1, 2.5)	0.640
Candy and chewables	475	6.7 (3.3–13.2)	6.4 (3–12.8)	−0.3 (−1.4, 0.7)	0.543
Desserts	187	70.4 (30.2–187.6)	40.2 (20.1–93.8)	−30.2 (−66.5, 6.2)	0.104
Chocolate candies	443	10.1 (5–23.5)	11.8 (5–23.5)	1.7 (0.2, 3.2)	0.028
Instant soups	72	15.0 (7.5–35.1)	22.5 (7.5–35.1)	7.5 (0.8, 14.2)	0.027
Processed meats	681	18.7 (8–37.4)	22.6 (10.7–37.7)	3.9 (0.6, 7.3)	0.022
Dairy desserts	543	25.4 (10.9–50.9)	15.5 (5.5–30.1)	−9.9 (−12.6, −7.2)	<0.001
Snacks	480	6.3 (2.7–12.6)	8.1 (2.7–12.6)	1.8 (−0.2, 3.8)	0.083

Note: Values are reported for participants who were consumers in both 2019 and 2021; therefore, the number of participants varies across categories. * *p*-value obtained from quantile regression comparing median daily intake between 2019 and 2021. CI = confidence interval. N.D.: not determined; for water energy content is 0 kcal.

**Table 5:** Per consumer daily nutrient intake (total sugar, sodium, saturated fatty acids) of HFSS packaged products in 2019 and 2021.

Beverage/Food Category	Consumers (n)	Total Sugars (g)				Sodium (mg)				Saturated Fatty Acids (g)			
		2019 Median (Q1–Q3)	2021 Median (Q1–Q3)	Median Difference 2021–2019 (95% CI)	*p*-Value *	2019 Median (Q1–Q3)	2021 Median (Q1–Q3)	Median Difference 2021–2019 (95% CI)	*p*-Value *	2019 Median (Q1–Q3)	2021 Median (Q1–Q3)	Median Difference 2021–2019 (95% CI)	*p*-Value *
Total food and beverages	1031	45.1 (27.1–70)	31.4 (20.2–50.7)	−13.6 (−16.1, −11.1)	<0.001	469.4 (306.4–690.2)	399.4 (260.5–579.7)	−70.0 (−92.5, −47.5)	<0.001	8.4 (5.2–13.4)	7.0 (4.2–11.2)	−1.3 (−1.8, −0.8)	<0.001
Total beverages	1030	28.7 (14.7–49.1)	21.5 (11.5–36.3)	−7.2 (−9.1, −5.2)	<0.001	270.3 (144.6–422.1)	203.5 (125.1–326.3)	−66.8 (−83.4, −50.2)	<0.001	4.9 (2.3–9.8)	3.7 (1.9–7.5)	−1.2 (−1.6, −0.8)	<0.001
Water	978	N.D.	N.D.	N.D.	N.D.	22.1 (13.9–35.7)	27.7 (15.6–41.6)	5.6 (3.9, 7.3)	<0.001	N.D.	N.D.	N.D.	N.D.
Nectars and flavored fruit drinks	475	1.9 (0.8–3.8)	1.5 (0.6–2.8)	−0.3 (−0.6, −0.1)	0.010	5.0 (2.1–11)	4.8 (2.1–9.3)	−0.2 (−1.0, 0.6)	0.598	N.D.	N.D.	N.D.	N.D.
Carbonated and sports drinks	572	1.7 (0.8–3.3)	1.7 (0.8–3.9)	0.1 (−0.1, 0.2)	0.581	2.5 (1.1–6.9)	2.5 (1.1–6.9)	0.0 (−0.4, 0.4)	1.000	N.D.	N.D.	N.D.	N.D.
Teas	792	0.1 (0–0.1)	0.1 (0–0.1)	0.0 (0.0, 0.0)	0.573	3.8 (1.9–9)	3.9 (2.5–9)	0.2 (−0.4, 0.7)	0.573	N.D.	N.D.	N.D.	N.D.
Milk	420	11.3 (4.8–22.7)	11.3 (4.8–22.7)	0.0 (−2.5, 2.5)	1.000	117.1 (49.2–234.2)	117.1 (49.2–234.2)	0.0 (−25.5, 25.5)	1.000	4.8 (2–9.6)	4.8 (2–9.6)	0.0 (−1.0, 1.0)	1.000
Dairy drinks	841	12.7 (5.3–35.3)	10.0 (4.4–21.9)	−2.7 (−4.2, −1.2)	<0.001	96.9 (42.9–267.3)	76.2 (35.1–153)	−20.7 (−30.9, −10.5)	<0.001	1.8 (0.9–3.7)	1.4 (0.6–2.7)	−0.4 (−0.6, −0.2)	<0.001
Formulas and supplements	50	7.2 (3.2–15)	7.2 (3–14.4)	0.0 (−3.3, 3.3)	1.000	84.1 (37.7–175.6)	84.1 (35.3–168.2)	0.0 (−38.8, 38.8)	1.000	1.9 (0.8–3.9)	1.9 (0.8–3.8)	0.0 (−0.9, 0.9)	1.000
Total food	1026	11.6 (5.8–21)	8.0 (4.1–15.4)	−3.6 (−4.4, −2.8)	<0.001	163.0 (93.3–267.6)	160.5 (87.1–285.2)	−2.5 (−15.3, 10.3)	0.702	2.8 (1.5–4.5)	2.4 (1.4–4.1)	−0.4 (−0.6, −0.2)	<0.001
Salty crackers	488	0.2 (0.1–0.4)	0.2 (0.1–0.4)	0.0 (0.0, 0.0)	1.000	33.3 (14.3–66.7)	33.3 (14.3–66.7)	0.0 (−3.8, 3.8)	1.000	0.2 (0.1–0.5)	0.2 (0.1–0.5)	0.0 (0.0, 0.0)	1.000
Sweet crackers	729	1.5 (1–3.2)	1.5 (1–3)	0.0 (−0.2, 0.2)	1.000	15.4 (9.9–33.1)	15.4 (9.9–30.9)	0.0 (−1.8, 1.8)	1.000	0.5 (0.3–1)	0.5 (0.3–0.9)	0.0 (0.0, 0.0)	1.000
Sweet pastries and bakery products	276	0.8 (0.4–1.7)	1.4 (0.7–2.1)	0.6 (0.4, 0.8)	<0.001	6.0 (2.6–12)	10.3 (5.2–15.5)	4.3 (2.7, 5.9)	<0.001	0.3 (0.1–0.6)	0.5 (0.2–0.7)	0.2 (0.1, 0.3)	<0.001
Breakfast cereals and bars	552	1.2 (0.5–2.1)	1.2 (0.5–2)	0.0 (−0.2, 0.1)	0.640	19.1 (8.2–29.4)	18.5 (6.8–29.4)	−0.7 (−3.4, 2.0)	0.625	0.0 (0–0.1)	0.0 (0–0)	0.0 (0.0, 0.0)	1.000
Candy and chewables	475	1.1 (0.5–2.2)	1.0 (0.5–2.1)	−0.1 (−0.2, 0.2)	0.543	1.3 (0.7–2.7)	1.3 (0.6–2.6)	−0.1 (−0.3, 0.1)	0.543	0.0 (0–0)	0.0 (0–0)	0.0 (0.0, 0.0)	0.543
Desserts	187	11.6 (5–30.8)	6.6 (3.3–15.4)	−5.0 (−10.9, 1.0)	0.104	31.7 (13.6–84.5)	18.1 (9.1–42.3)	−13.6 (−30.0, 2.8)	0.104	0.9 (0.4–2.3)	0.5 (0.2–1.2)	−0.4 (−0.8, 0.1)	0.104
Chocolate candies	443	0.8 (0.4–1.9)	1.0 (0.4–1.9)	0.1 (0.0, 0.2)	0.028	1.3 (0.7–3.1)	1.5 (0.7–3.1)	0.2 (0.0, 0.4)	0.028	0.4 (0.2–0.8)	0.4 (0.2–0.8)	0.1 (0.0, 0.1)	0.028
Instant soups	72	0.1 (0–0.2)	0.1 (0–0.2)	0.0 (0.0, 0.1)	0.027	107.9 (53.9–251.7)	161.8 (53.9–251.7)	53.9 (6.2, 101.7)	0.027	0.3 (0.2–0.8)	0.5 (0.2–0.8)	0.2 (0.0, 0.3)	0.027
Processed meats	681	0.0 (0–0.1)	0.1 (0–0.1)	0.0 (0.0, 0.0)	0.022	64.2 (27.5–128.3)	77.7 (36.7–129.2)	13.5 (1.9, 25.2)	0.022	0.5 (0.2–1)	0.6 (0.3–1)	0.1 (0.0, 0.2)	0.022
Dairy desserts	543	3.3 (1.4–6.6)	1.9 (0.7–3.7)	−1.3 (−1.7, −1.0)	<0.001	13.7 (5.9–27.3)	7.9 (2.9–14.8)	−5.8 (−7.2, −4.4)	<0.001	0.5 (0.2–1)	0.3 (0.1–0.6)	−0.2 (−0.2, −0.1)	<0.001
Snacks	480	0.0 (0–0)	0.0 (0–0)	0.0 (0.0, 0.0)	0.083	7.8 (3.3–15.6)	10.0 (3.3–15.6)	2.2 (−0.3, 4.8)	0.083	0.1 (0.1–0.3)	0.2 (0.1–0.3)	0.0 (0.0, 0.1)	0.083

Note: Values are reported for participants who were consumers in both 2019 and 2021; therefore, the number of participants varies across categories. * *p*-value obtained from quantile regression comparing median daily intake between 2019 and 2021. N.D.: not determined; nutrient not declared in the nutrition facts panel and therefore not estimated.

## Data Availability

The data presented in this study is available upon request from the corresponding author.

## Supplementary Materials

The following supporting information can be downloaded at: https://www.mdpi.com/article/10.3390/nu18213504/s1. Supplementary Material S1: Food Frequency Questionnaire (FFQ) and Food Portion Photo Atlas. Supplementary Table S1: Sociodemographic characteristics of mothers and children who did and did not partipcate in the follow-up survey in 2021; Supplementary Table S2: Spearman correlations between the Food Frequency Questionnaire and 24-h dietary recalls for HFSS package products intake and nutrient estimates (*n* = 118); Supplementary Table S3: Adjusted Spearman correlations between the Food Frequency Questionnaire and 24-h dietary recalls for HFSS package products intake and nutrient estimates (*n* = 118); Supplementary Table S4: Per consumer daily intake of HFSS packaged foods (grams) and beverages (milliliters), stratified by socioeconomic level in 2019 and 2021.

## Abbreviations

The following abbreviations are used in this manuscript:

NCD	Non-communicable disease
UPF	Ultra-processed food
PAHO	Pan American Health Organization
FOPWL	Front-of-package warning label
FFQ	Food Frequency Questionnaire
SEL	Socioeconomic level

## Appendix A

### Sampling Procedure and Sample Size Calculation

A stratified sampling method was employed to ensure equal representation of socioeconomic levels (SEL) B (mid-high) and D (mid-low) across the four selected districts, with 50% of the sample allocated to each group. The SEL classification of each block was determined according to the “Stratified Plans of Metropolitan Lima at the Block Level by Household Per Capita Income,” developed by the National Institute of Statistics and Informatics [19]. Briefly, this method combines information from a survey (National Household Survey) with census data (District Population and Housing Census), in addition to other administrative sources, to estimate household per capita income at the block level. Further methodological details are available in the INEI report [19].

The sample size for the panel study was calculated using G*Power 3.1 software for a repeated measures analysis with two between-subject groups (SEL B and SEL D). Parameters included and effect size of 0.1, a statistical power of 99%, a confidence level of 99%, and an assumed correlation of 0.25 between measurements. Based on these specifications, a minimum of 906 participants was required. To account for an estimated 25% loss to follow-up, a total of 1200 participants were initially considered for inclusion.

Sampling was conducted in three stages. In the first stage, city blocks were selected using geographic codes provided by the National Institute of Statistics and Informatics (INEI) based on the digital cartography of the 2017 Census of Lima and Callao. In the second stage, fieldworkers conducted systematic household visits within the selected blocks, following a clockwise pattern. Each visit began at the first household, eligibility was assessed, and if a household did not meet inclusion criteria (e.g., no children in the target age range, mother declined participation, household vacant or unavailable), the next eligible household was selected. This process continued until all households in the block had been screened. If the required number of participants was not reached within a block, the next adjacent block was selected, maintaining a clockwise pattern until the target sample size for each district was achieved.

In the final stage, participants were selected using predefined gender quotas for each primary sampling unit. In households with more than one eligible mother or more than one child within the age range, the child with the most recent birthday was selected.
