# Supplementary material for: Changes in Children’s Dietary Intake of Selected Packaged Products Between 2019 and 2021 in the Context of Peru’s Front-of-Package Warning Label Policy: A Longitudinal Cohort Study
Source: Nutrients. 2026 Oct 25;18(21):3504. doi: 10.3390/nu18213504 (PMC13764887; doi:10.3390/nu18213504)
Supplement: Supplementary file 1 [file nutrients-18-03504-s001.zip › nutrients-4603695-supplementary.pdf]

Supplementary Materials

Supplementary Material S1. Food Frequency Questionnaire (FFQ) and Food Portion Photo Atlas

**FOOD FREQUENCY QUESTIONNAIRE**			
Say to the participant:<br>*Now I will ask you about a list of beverages to find out whether (child's name) has consumed them, how often, and in what quantity. Please answer based on the consumption during the last 30 days. We ask you to be sincere and precise with your answers—there are no right or wrong answers.*			
Say to participant: First, I will ask about tap water, boiled or unboiled			
1	Has (child's name) consumed tap water, boiled or unboiled, in the last 30 days?	1	No ***(Skip to question 6)***
		2	Yes
2	How often did (child's name) drink tap water, boiled or unboiled?<br>***Reed the options. Mark only one.***	1	Monthly
		2	Weekly
		3	Daily
3	How many times per (month/week/day) did (child's name) drink tap water, boiled or unboiled?		
4	(Show the picture) Each time (child's name) drinks tap water, boiled or unboiled, what type of container is used? Please point to the one most similar to what is usually used.	1	1 bottle of water (600–635 mL)
		2	1 bottle of beverage (e.g., Tampico, Aquarius, or Sporade – 500 mL)
		3	1 glass or half bottle (200–250 mL)
		4	1 juice box (235 mL)
		5	1 small yogurt bottle (180 mL)
		6	1 small juice box or yogurt cup (120–145 mL)
5	How many of these containers are consumed each time (child's name) drinks tap water, boiled or unboiled?		
Say to participant: Now I will ask you about Bottled or jug water, with or without gas			
6	Has (child's name) drunk Bottled or jug water, with or without gas, in the last 30 days?	1	No ***(Skip to question 12)***
		2	Yes
7	How often did (child's name) drink Bottled or jug water, with or without gas?<br>***Reed the options. Mark only one.***	1	Monthly
		2	Weekly
		3	Daily
8	How many times per (month/week/day) did (child's name) drink Bottled or jug water, with or without gas?		

Código de la participante	☐☐☐☐☐ ☐☐	Código de la encuestadora	☐☐

9	***(Show the picture sheet.) Each time (child's name) drinks*** Bottled or jug water, with or without gas, what type of container is used?	1	1 bottle of water (600–635 mL)
		2	1 bottle of beverage (e.g., Tampico, Aquarius, or Sporade – 500 mL)
		3	1 glass or half bottle (200–250 mL)
		4	1 juice box (235 mL)
		5	1 small yogurt bottle (180 mL)
		6	1 small juice box or yogurt cup (120–145 mL)
10	How many of these containers does (child's name) drink each time he/she consumes bottled water?		
11	What brands does (child's name) usually drink?<br>***Do not read options***<br>***Mark all that apply***	1	Cielo
		2	San Luis
		3	San Mateo
		4	Other ***(specify)***:
Say to participant: Now I will ask you about 100% natural juices prepared at home			
12	Has (child's name) drunk 100% natural juices prepared at home, in the last 30 days?	1	No ***(Skip to question 18)***
		2	Yes
13	How often did (child's name) drink 100% natural juices prepared at home?<br>***Reed the options. Mark only one.***	1	Monthly
		2	Weekly
		3	Daily
14	How many times per (month/week/day) did (child's name) drink 100% natural juices prepared at home?		
15	***(Show the picture sheet.) Each time (child's name) drinks*** 100% natural juices prepared at home, what type of container is used? Please point to the one most similar to what is usually used.	1	1 bottle of water (600–635 mL)
		2	1 bottle of beverage (e.g., Tampico, Aquarius, or Sporade – 500 mL)
		3	1 glass or half bottle (200–250 mL)
		4	1 juice box (235 mL)
		5	1 small yogurt bottle (180 mL)
		6	1 small juice box or yogurt cup (120–145 mL)
16	How many of these containers does (child's name) drink each time he/she consumes 100% natural juices ?		
17	Do you add sugar or honey?	1	No
		2	Yes
Say to participant: Now I will ask you about Homemade fruit drinks such as apple water, starfruit water, lemonade, or purple corn drink (chicha morada)			
18	Has (child's name) drunk Homemade fruit drinks such as apple water, starfruit water,	1	No ***(Skip to question 24)***

	lemonade, or purple corn drink (chicha morada), in the last 30 days?	2	Yes

19	How often did (child's name) drink Homemade fruit drinks such as apple water, starfruit water, lemonade, or purple corn drink (chicha morada)?<br>***Reed the options. Mark only one.***	1	Monthly
		2	Weekly
		3	Daily
20	How many times per (month/week/day) did (child's name) drink Homemade fruit drinks such as apple water, starfruit water, lemonade, or purple corn drink (chicha morada)?		
21	***(Show the picture sheet.) Each time (child's name) drinks*** Homemade fruit drinks such as apple water, starfruit water, lemonade, or purple corn drink (chicha morada), what type of container is used? Please point to the one most similar to what is usually used.	1	1 bottle of water (600–635 mL)
		2	1 bottle of beverage (e.g., Tampico, Aquarius, or Sporade – 500 mL)
		3	1 glass or half bottle (200–250 mL)
		4	1 juice box (235 mL)
		5	1 small yogurt bottle (180 mL)
		6	1 small juice box or yogurt cup (120–145 mL)
22	How many of these containers does (child's name) drink each time he/she consumes homemade fruit drinks?		
23	Do you add sugar or honey?	1	No
		2	Yes
Say to participant: Now I will ask you about <u>Packaged nectars or boxed juices</u>			
24	Has (child's name) drunk Packaged nectars or boxed juices, in the last 30 days?	1	No ***(Skip to question 30)***
		2	Yes
25	How often did (child's name) drink Packaged nectars or boxed juices?<br>***Reed the options. Mark only one.***	1	Monthly
		2	Weekly
		3	Daily
26	How many times per (month/week/day) did (child's name) drink Packaged nectars or boxed juices?		
27	***(Show the picture sheet.)*** Each time (child's name) drinks Packaged nectars or boxed juices, what type of container is used? Please point to the one most similar to what is usually used.	1	1 bottle of water (600–635 mL)
		2	1 bottle of beverage (e.g., Tampico, Aquarius, or Sporade – 500 mL)
		3	1 glass or half bottle (200–250 mL)
		4	1 juice box (235 mL)
		5	1 small yogurt bottle (180 mL)
		6	1 small juice box or yogurt cup (120–145 mL)
28	How many of these containers does (child's name) drink each time he/she consumes Packaged nectars or boxed juices?		

Código de la participante	☐☐☐☐☐ ☐☐	Código de la encuestadora	☐☐

29	What brands does (child's name) usually drink?<br>***<u>Do not read options</u>***<br>***Mark all that apply***	1	Pulp
		2	Frugos
		3	Gloria
		4	Other ***(specify)***:
Say to participant: Now I will ask you about <u>Artificial drinks, either ready-to-drink or powdered.</u>			
30	Has (child's name) drunk Artificial drinks, either ready-to-drink or powdered., in the last 30 days?	1	No ***(Skip to question 36)***
		2	Yes
31	How often did (child's name) drink Artificial drinks, either ready-to-drink or powdered.?<br>***Reed the options. Mark only one.***	1	Monthly
		2	Weekly
		3	Daily
32	How many times per (month/week/day) did (child's name) drink Artificial drinks, either ready-to-drink or powdered.?		
33	***(Show the picture sheet)*** Each time (child's name) drinks Artificial drinks, either ready-to-drink or powdered, what type of container is used? Please point to the one most similar to what is usually used.	1	1 bottle of water (600–635 mL)
		2	1 bottle of beverage (e.g., Tampico, Aquarius, or Sporade – 500 mL)
		3	1 glass or half bottle (200–250 mL)
		4	1 juice box (235 mL)
		5	1 small yogurt bottle (180 mL)
		6	1 small juice box or yogurt cup (120–145 mL)
34	How many of these containers does (child's name) drink each time he/she consumes artificial drinks?		
35	What brands does (child's name) usually drink?<br>***<u>Do not read options</u>***<br>***Mark all that apply***	1	Cifrut
		2	Tampico
		3	Aquarius
		4	Negrita
		5	Zuko
		6	Other ***(specify)***:
Say to participant: Now I will ask you about <u>Herbal infusions, tea, or emoliente (herbal beverage).</u>			
36	Has (child's name) drunk Herbal infusions, tea, or emoliente (herbal beverage)., in the last 30 days?	1	No ***(Skip to question 42)***
		2	Yes
37	How often did (child's name) Herbal infusions, tea, or emoliente ?<br>***Reed the options. Mark only one.***	1	Monthly
		2	Weekly
		3	Daily
38	How many times per (month/week/day) did (child's name) drink Herbal infusions, tea, or emoliente?		

39	(***Show the picture sheet.)*** Each time (child's name) drinks infusions, tea, or emoliente, what type of container is used? Please point to the one most similar to what is usually used.	1	1 bottle of water (600–635 mL)
		2	1 bottle of beverage (e.g., Tampico, Aquarius, or Sporade – 500 mL)
		3	1 glass or half bottle (200–250 mL)
		4	1 juice box (235 mL)
		5	1 small yogurt bottle (180 mL)
		6	1 small juice box or yogurt cup (120–145 mL)
40	How many of these containers does (child's name) drink each time he/she consumes infusions or emoliente?		
41	Do you add sugar or honey?	1	No
		2	Yes
Say to participant: Now I will ask you about Milk without flavor (blue, red, or green label), whether canned, boxed, bagged, or powder milk.			
42	Has (child's name) drunk Plain milk (whole, light, or lactose-free), in the last 30 days?	1	No ***(Skip to question 48)***
		2	Yes
43	How often did (child's name) drink Plain milk (whole, light, or lactose-free)?<br>***Reed the options. Mark only one.***	1	Monthly
		2	Weekly
		3	Daily
44	How many times per (month/week/day) did (child's name) drink plain milk (whole, light, or lactose-free) canned, boxed, bagged or powder milk?		
45	***(Show the picture sheet.)*** Each time (child's name) drinks plain milk (whole, light, or lactose-free), what type of container is used? Please point to the one most similar to what is usually used.	1	1 bottle of water (600–635 mL)
		2	1 bottle of beverage (e.g., Tampico, Aquarius, or Sporade – 500 mL)
		3	1 glass or half bottle (200–250 mL)
		4	1 juice box (235 mL)
		5	1 small yogurt bottle (180 mL)
		6	1 small juice box or yogurt cup (120–145 mL)
46	How many of these containers does (child's name) drink each time he/she consumes whole milk?		
47	Do you add sugar or honey?	1	No
		2	Yes
Say to participant: Now I will ask you about Flavored milk, such as chocolate, vanilla, or strawberry milk (e.g., Milo, Nesquik)			
48	Has (child's name) drunk flavored milk, in the last 30 days?	1	No ***(Skip to question 55)***
		2	Yes

49	How often did (child's name) drink flavored milk?<br>***Reed the options. Mark only one.***	1	Monthly
		2	Weekly
		3	Daily
50	How many times per (month/week/day) did (child's name) drink flavored milk?		
51	***(Show the picture sheet.)*** Each time (child's name) drinks flavored milk, what type of container is used? Please point to the one most similar to what is usually used.	1	1 bottle of water (600–635 mL)
		2	1 bottle of beverage (e.g., Tampico, Aquarius, or Sporade – 500 mL)
		3	1 glass or half bottle (200–250 mL)
		4	1 juice box (235 mL)
		5	1 small yogurt bottle (180 mL)
		6	1 small juice box or yogurt cup (120–145 mL)
52	How many of these containers does (child's name) drink each time he/she consumes flavored milk?		
53	Do you add sugar or honey?	1	No
		2	Yes
54	What brands does (child's name) usually drink?<br>***Do not read options***<br>***Mark all that apply***	1	Laive Chocolate Milk
		2	Gloria Chocolate Milk
		3	Milo
		4	Nesquik
		5	Other ***(specify)***:
Say to participant: Now I will ask you about drinkable yogurt			
55	Has (child's name) drunk Drinkable yogurt in the last 30 days?	1	No ***(Skip to question 61)***
		2	Yes
56	How often did (child's name) drink drinkable yogurt?<br>***Reed the options. Mark only one.***	1	Monthly
		2	Weekly
		3	Daily
57	How many times per (month/week/day) did (child's name) drink drinkable yogurt?		
58	***(Show the picture sheet.)*** Each time (child's name) drinks drinkable yogurt, what type of container is used? Please point to the one most similar to what is usually used.	1	1 bottle of water (600–635 mL)
		2	1 bottle of beverage (e.g., Tampico, Aquarius, or Sporade – 500 mL)
		3	1 glass or half bottle (200–250 mL)
		4	1 juice box (235 mL)
		5	1 small yogurt bottle (180 mL)
		6	1 small juice box or yogurt cup (120–145 mL)

59	How many of these containers does (child's name) drink each time he/she consume drinkable yogurt?		
60	What brands does (child's name) usually drink?<br>***<u>Do not read options</u>***<br>***Mark all that apply***	1	Gloria
		2	Laive
		3	Gloria Slim (light)
		4	Laive Sbelt (light)
		5	Other ***(specify)***:
Say to participant: Now I will ask you about <u>sodas</u>			
61	Has (child's name) drunk Sodas in the last 30 days?	1	No ***(Skip to question 67)***
		2	Yes
62	How often did (child's name) drink Sodas?<br>***Reed the options. Mark only one.***	1	Monthly
		2	Weekly
		3	Daily
63	How many times per (month/week/day) did (child's name) drink Sodas?		
64	***(Show the picture sheet.) Each time (child's name) drinks*** Sodas, what type of container is used? Please point to the one most similar to what is usually used.	1	1 bottle of water (600–635 mL)
		2	1 bottle of beverage (e.g., Tampico, Aquarius, or Sporade – 500 mL)
		3	1 glass or half bottle (200–250 mL)
		4	1 juice box (235 mL)
		5	1 small yogurt bottle (180 mL)
		6	1 small juice box or yogurt cup (120–145 mL)
65	How many of these containers does (child's name) drink each time he/she consumes Sodas?		
66	What brands does (child's name) usually drink?<br>***<u>Do not read options</u>***<br>***Mark all that apply***	1	Coca Cola
		2	Inka Cola
		3	Fanta
		4	Pepsi
		5	Kola Real
		6	Coca Cola sin azúcar
		7	Inka Cola sin azúcar
		8	Pepsi zero
		9	Other ***(specify)***:
Say to participant: Now I will ask you about <u>Sports or rehydration drinks</u>			
67	Has (child's name) drunk Sports or rehydration drinks in the last 30 days?	1	No ***(Skip to question 73)***
		2	Yes

68	How often did (child's name) drink Sports or rehydration drinks?<br>***Reed the options. Mark only one.***	1	Monthly
		2	Weekly
		3	Daily
69	How many times per (month/week/day) did (child's name) drink Sports or rehydration drinks?		
70	***(Show the picture sheet.)*** Each time (child's name) drinks Sports or rehydration drinks, what type of container is used? Please point to the one most similar to what is usually used.	1	1 bottle of water (600–635 mL)
		2	1 bottle of beverage (e.g., Tampico, Aquarius, or Sporade – 500 mL)
		3	1 glass or half bottle (200–250 mL)
		4	1 juice box (235 mL)
		5	1 small yogurt bottle (180 mL)
		6	1 small juice box or yogurt cup (120–145 mL)
71	How many of these containers does (child's name) drink each time he/she consume sports or rehydration drinks?		
72	What brands does (child's name) usually drink?<br>***<u>Do not read options</u>***<br>***Mark all that apply***	1	Gatorade
		2	Sporade
		3	Other ***(specify)***:
Say to participant: Now I will ask you about <u>Cereal-based beverages, such as oatmeal drinks, quinoa drinks, maca drinks, or "7 seeds" mixtures</u>			
73	Has (child's name) drunk Cereal-based beverages, such as oatmeal drinks, quinoa drinks, maca drinks, or "7 seeds" mixtures in the last 30 days?	1	No ***(Skip to question 79)***
		2	Yes
74	How often did (child's name) drink Cereal-based beverages, such as oatmeal drinks, quinoa drinks, maca drinks, or "7 seeds" mixtures?<br>***Reed the options. Mark only one.***	1	Monthly
		2	Weekly
		3	Daily
75	How many times per (month/week/day) did (child's name) drink Cereal-based beverages, such as oatmeal drinks, quinoa drinks, maca drinks, or "7 seeds" mixtures?		
76	***(Show the picture sheet.)*** Each time (child's name) drinks Cereal-based beverages, such as oatmeal drinks, quinoa drinks, maca drinks, or "7 seeds" mixtures, what type of container is used? Please point to the one most similar to what is usually used.	1	1 bottle of water (600–635 mL)
		2	1 bottle of beverage (e.g., Tampico, Aquarius, or Sporade – 500 mL)
		3	1 glass or half bottle (200–250 mL)
		4	1 juice box (235 mL)
		5	1 small yogurt bottle (180 mL)
		6	1 small juice box or yogurt cup (120–145 mL)
77	How many of these containers does (child's name) drink each time he/she consumes cereal-based beverages?		
78	Do you add sugar or honey?	1	No

		2	Yes

Say to participant: Now I will ask you about <u>Formulas or nutritional supplements</u>			
79	Has (child's name) drunk Formulas or nutritional supplements in the last 30 days?	1	No ***(Skip to question 84)***
		2	Yes
80	How often did (child's name) drink Formulas or nutritional supplements?<br>***Reed the options. Mark only one.***	1	Monthly
		2	Weekly
		3	Daily
81	How many times per (month/week/day) did (child's name) drink Formulas or nutritional supplements?		
82	***(Show the picture sheet.)*** Each time (child's name) drinks Formulas or nutritional supplements, what type of container is used? Please point to the one most similar to what is usually used.	1	1 bottle of water (600–635 mL)
		2	1 bottle of beverage (e.g., Tampico, Aquarius, or Sporade – 500 mL)
		3	1 glass or half bottle (200–250 mL)
		4	1 juice box (235 mL)
		5	1 small yogurt bottle (180 mL)
		6	1 small juice box or yogurt cup (120–145 mL)
83	How many of these containers does (child's name) drink each time he/she consumes formulas or nutritional supplements?		
Say to participant: Now I will ask you about foods that *(child's name)* has eaten during the last 30 days, and how often.			
Say to participant: First I will ask about <u>Cakes or bakery products</u>			
84	Has (child's name) eaten Cakes or bakery products in the last 30 days?	1	No ***(Skip to question 88)***
		2	Yes
85	How often did (child's name) eat Cakes or bakery products?<br>***Reed the options. Mark only one.***	1	Monthly
		2	Weekly
		3	Daily
86	How many times per (month/week/day) did (child's name) eat Cakes or bakery products?		
87	***Show the picture sheet.) Each time (child's name) eats*** Cakes or bakery products, how many portions like this does he/she eat?<br>***(Consider that one slice is equivalent to two small muffins.)***		

**Código de la participante**	☐	☐	☐	☐	☐	☐ ☐	**Código de la encuestadora**	☐ ☐

Say to participant: Now I will ask you about Sweet cookies (with filling or coated in chocolate)			
88	Has (child's name) eaten Sweet cookies (with filling or coated in chocolate) in the last 30 days?	1	No ***(Skip to question 92)***
		2	Yes
89	How often did (child's name) eat Sweet cookies (with filling or coated in chocolate)? ***Reed the options. Mark only one.***	1	Monthly
		2	Weekly
		3	Daily
90	How many times per (month/week/day) did (child's name) eat Sweet cookies (with filling or coated in chocolate)?		
91	***Show the picture sheet.)*** Each time (child's name) eats Sweet cookies (with filling or coated in chocolate), how many portions like this does he/she eat?		
Say to participant: Now I will ask you about Crackers			
92	Has (child's name) eaten Crackers in the last 30 days?	1	No ***(Skip to question 96)***
		2	Yes
93	How often did (child's name) eat Crackers? ***Reed the options. Mark only one.***	1	Monthly
		2	Weekly
		3	Daily
94	How many times per (month/week/day) did (child's name) eat Crackers?		
95	***Show the picture sheet.)*** Each time (child's name) eats Crackers, how many packages like this does he/she eat?		
Say to participant: Now I will ask you about Whole grain cookies			
96	Has (child's name) eaten Whole grain cookies in the last 30 days?	1	No ***(Skip to question 100)***
		2	Yes
97	How often did (child's name) eat Whole grain cookies? ***Reed the options. Mark only one.***	1	Monthly
		2	Weekly
		3	Daily
98	How many times per (month/week/day) did (child's name) eat Whole grain cookies?		
99	***Show the picture sheet.)*** Each time (child's name) eats Whole grain cookies, how many packages like this does he/she eat?		

Say to participant: Now I will ask you about <u>Ice creams</u>			
100	Has (child's name) eaten Ice creams in the last 30 days?	1	No ***(Skip to question 104)***
		2	Yes
101	How often did (child's name) eat Ice creams?<br>***Reed the options. Mark only one.***	1	Monthly
		2	Weekly
		3	Daily
102	How many times per (month/week/day) did (child's name) eat Ice creams?		
103	***Show the picture sheet.) Each time (child's name) eats*** Ice creams, how many portions like this does he/she eat?<br>***(Consider that one popsicle equals one scoop of ice cream)***		
Say to participant: Now I will ask you about <u>Chocolates</u>			
104	Has (child's name) eaten Chocolates in the last 30 days?	1	No ***(Skip to question 108)***
		2	Yes
105	How often did (child's name) eat Chocolates?<br>***Reed the options. Mark only one.***	1	Monthly
		2	Weekly
		3	Daily
106	How many times per (month/week/day) did (child's name) eat Chocolates?		
107	***Show the picture sheet.) Each time (child's name) eats*** Chocolates, how many bars like this does he/she eat?		
Say to participant: Now I will ask you about <u>Candies, toffees, or gummies</u>			
108	Has (child's name) eaten Candies, toffees, or gummies in the last 30 days?	1	No ***(Skip to question 112)***
		2	Yes
109	How often did (child's name) eat Candies, toffees, or gummies?<br>***Reed the options. Mark only one.***	1	Monthly
		2	Weekly
		3	Daily
110	How many times per (month/week/day) did (child's name) eat Candies, toffees, or gummies?		
111	***Show the picture sheet.)*** Each time (child's name) eats Candies, toffees, or gummies,<br>how many portions like this does he/she eat?<br>***Consider individual pieces as one unit.***		

Código de la participante	☐☐☐☐☐ ☐☐	Código de la encuestadora	☐☐

Say to participant: Now I will ask you about Lollipops			
112	Has (child's name) eaten Lollipops in the last 30 days?	1	No ***(Skip to question 116)***
		2	Yes
113	How often did (child's name) eat Lollipops?<br>***Reed the options. Mark only one.***	1	Monthly
		2	Weekly
		3	Daily
114	How many times per (month/week/day) did (child's name) eat Lollipops?		
115	***Show the picture sheet.)*** Each time (child's name) eats Lollipops, how many lollipops like this does he/she eat?		
Say to participant: Now I will ask you about Yogurt with cereal (ready to mix or "batimix")			
116	Has (child's name) eaten Yogurt with cereal (ready to mix or "batimix") in the last 30 days?	1	No ***(Skip to question 120)***
		2	Yes
117	How often did (child's name) eat Yogurt with cereal (ready to mix or "batimix")?<br>***Reed the options. Mark only one.***	1	Monthly
		2	Weekly
		3	Daily
118	How many times per (month/week/day) did (child's name) eat Yogurt with cereal (ready to mix or "batimix")?		
119	***Show the picture sheet.)*** Each time (child's name) eats Yogurt with cereal (ready to mix or "batimix"), how many cups like this does he/she eat?		
Say to participant: Now I will ask you about Jelly			
120	Has (child's name) eaten Jelly in the last 30 days?	1	No ***(Skip to question 124)***
		2	Yes
121	How often did (child's name) eat Jelly ?<br>***Reed the options. Mark only one.***	1	Monthly
		2	Weekly
		3	Daily
122	How many times per (month/week/day) did (child's name) eat Jelly?		
123	***Show the picture sheet.)*** Each time (child's name) eats Gelatin, how many portions like this does he/she eat?<br>***Consider one small gelatin cup as one serving***		

Say to participant: Now I will ask you about Breakfast cereals no added sugar			
124	Has (child's name) eaten Breakfast cereals no added sugar in the last 30 days?	1	No ***(Skip to question 128)***
		2	Yes
125	How often did (child's name) eat Breakfast cereals no added sugar?<br>***Reed the options. Mark only one.***	1	Monthly
		2	Weekly
		3	Daily
126	How many times per (month/week/day) did (child's name) eat Breakfast cereals no added sugar?		
127	***Show the picture sheet.)*** Each time (child's name) eats Breakfast cereals no added sugar, how many small bags or handfulslike this does he/she eat?<br>***Consider one small bag = one handful***		
Say to participant: Now I will ask you about Sweetened breakfast cereals (with sugar, chocolate, or flavored)			
128	Has (child's name) eaten Sweetened breakfast cereals (with sugar, chocolate, or flavored) in the last 30 days?	1	No ***(Skip to question 132)***
		2	Yes
129	How often did (child's name) eat Sweetened breakfast cereals (with sugar, chocolate, or flavored)?<br>***Reed the options. Mark only one.***	1	Monthly
		2	Weekly
		3	Daily
130	How many times per (month/week/day) did (child's name) eat Sweetened breakfast cereals (with sugar, chocolate, or flavored)?		
131	***Show the picture sheet.) Each time (child's name) eats*** Sweetened breakfast cereals (with sugar, chocolate, or flavored),<br>how many small bags or handfulslike this does he/she eat?<br>***Consider one small bag = one handful***		
Say to participant: Now I will ask you about Cereal bars			
132	Has (child's name) eaten Cereal bars in the last 30 days?	1	No ***(Skip to question 136)***
		2	Yes
133	How often did (child's name) eat Cereal bars?<br>***Reed the options. Mark only one.***	1	Monthly
		2	Weekly
		3	Daily

134	How many times per (month/week/day) did (child's name) eat Cereal bars?		
135	***Show the picture sheet.)*** Each time (child's name) eats Cereal bars, how many bars like this does he/she eat?		
Say to participant: Now I will ask you about Salty snacks (chips, Doritos, Cheetos)			
136	Has (child's name) eaten Salty snacks (chips, Doritos, Cheetos) in the last 30 days?	1	No ***(Skip to question 140)***
		2	Yes
137	How often did (child's name) eat Salty snacks (chips, Doritos, Cheetos)?<br>***Reed the options. Mark only one.***	1	Monthly
		2	Weekly
		3	Daily
138	How many times per (month/week/day) did (child's name) eat Salty snacks (chips, Doritos, Cheetos)?		
139	***Show the picture sheet.) Each time (child's name) eats*** Salty snacks (chips, Doritos, Cheetos), how many portions like this does he/she eat?<br>***Consider one unit as one small bag***		
Say to participant: Now I will ask you about Hot dog, processed ham			
140	Has (child's name) eaten Hot dog or processed ham in the last 30 days?	1	No ***(Skip to question 144)***
		2	Yes
141	How often did (child's name) eat Hot dog or processed ham?<br>***Reed the options. Mark only one.***	1	Monthly
		2	Weekly
		3	Daily
142	How many times per (month/week/day) did (child's name) eat Hot dog or processed ham?		
143	***Show the picture sheet.) Each time (child's name) eats*** Hot dog or processed jam, how many portions like this does he/she eat?<br>***Consider one hot dog = two slices of ham***		
Say to participant: Now I will ask you about Instant soups			
144	Has (child's name) eaten Instant soups in the last 30 days?	1	No ***(Pase a la pregunta 148)***
		2	Yes

145	How often did (child’s name) eat Instant soups?<br>***Reed the options. Mark only one.***	1	Monthly
		2	Weekly
		3	Daily
146	How many times per (month/week/day) did (child’s name) eat Instant soups?		
147	***Show the picture sheet.) Each time (child’s name) eats*** Instant soups,<br>how many bowls like this does he/she eat?<br>***Consider one unit as one bowl***		
Say to participant: Now I will ask you about <u>Nuggets</u>			
148	Has (child’s name) eaten Nuggets in the last 30 days?	1	No
		2	Yes
149	How often did (child’s name) eat Nuggets ?<br>***Reed the options. Mark only one.***	1	Monthly
		2	Weekly
		3	Daily
150	How many times per (month/week/day) did (child’s name) eat Nuggets?		
151	***Show the picture sheet.) Each time (child’s name) eats*** Nuggets,<br>how many pairs like this does he/she eat?<br>***Consider one unit as two nuggets***		

BEVERAGES

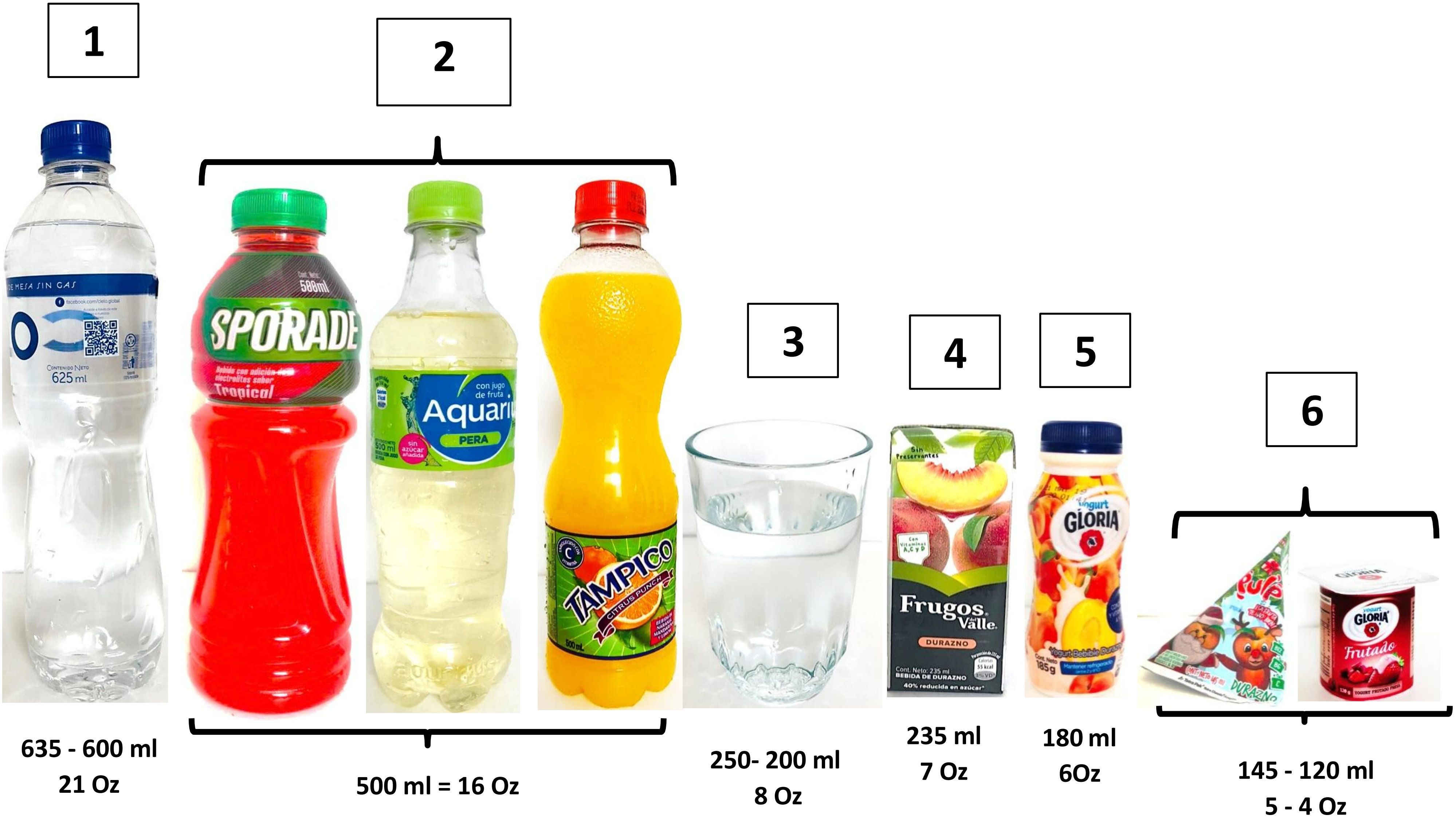
1
2
3
4
5
6
625 ml
SPORADE
Tropical
500ml
Aquari
con jugo de fruta
PERA
TAMPICO
CITRUS PUNCH
Frugos del Valle
DURAZNO
GLORIA
Frutado
635 - 600 ml
21 Oz
500 ml = 16 Oz
250- 200 ml
8 Oz
235 ml
7 Oz
180 ml
6Oz
145 - 120 ml
5 - 4 Oz

1. CAKES AND BAKERY PRODUCTS

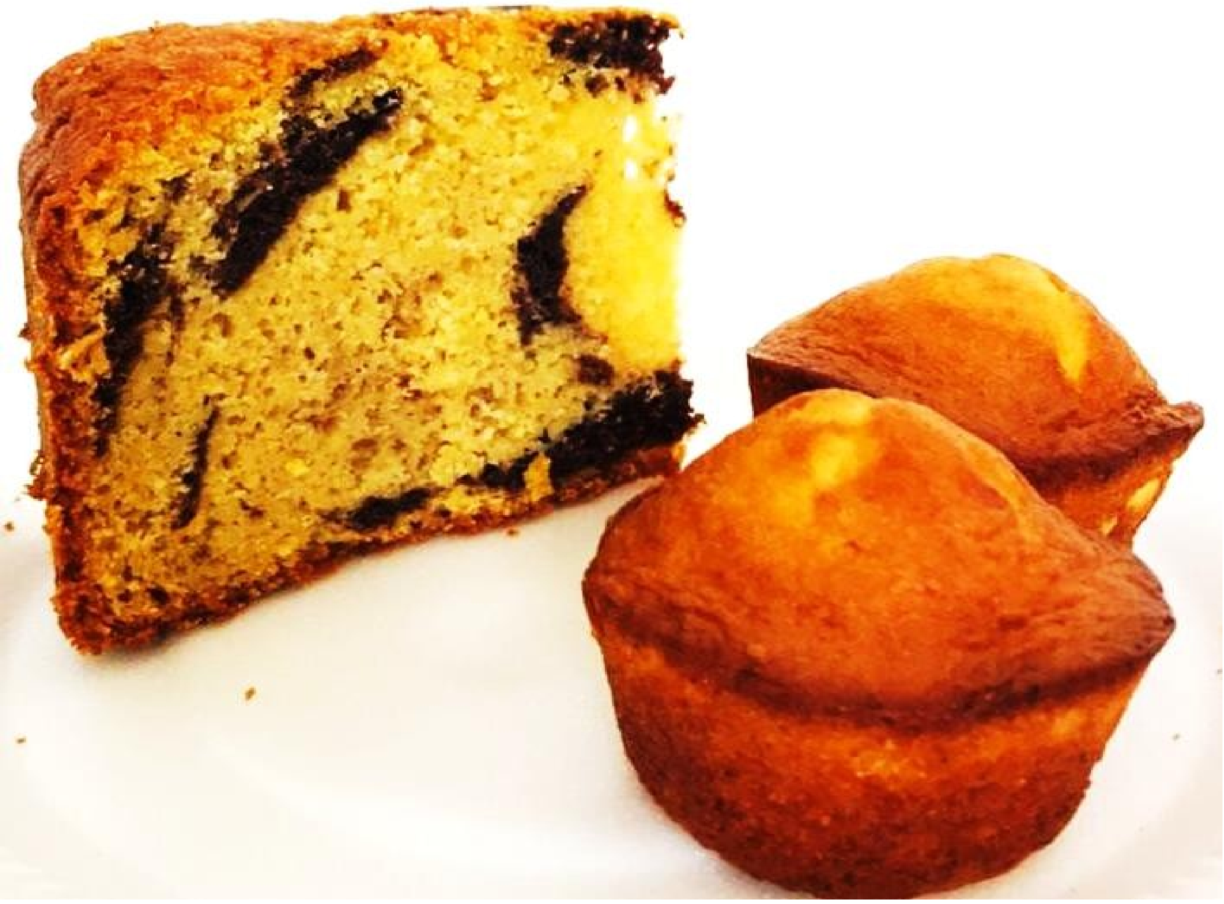

1 slice of round cake or 2 “Bimboletes” mini cakes

2. SWEET COOKIES

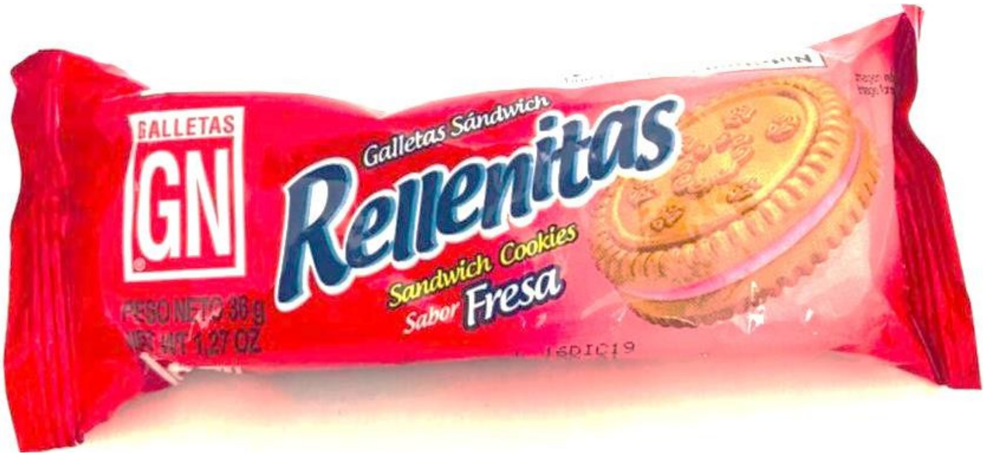

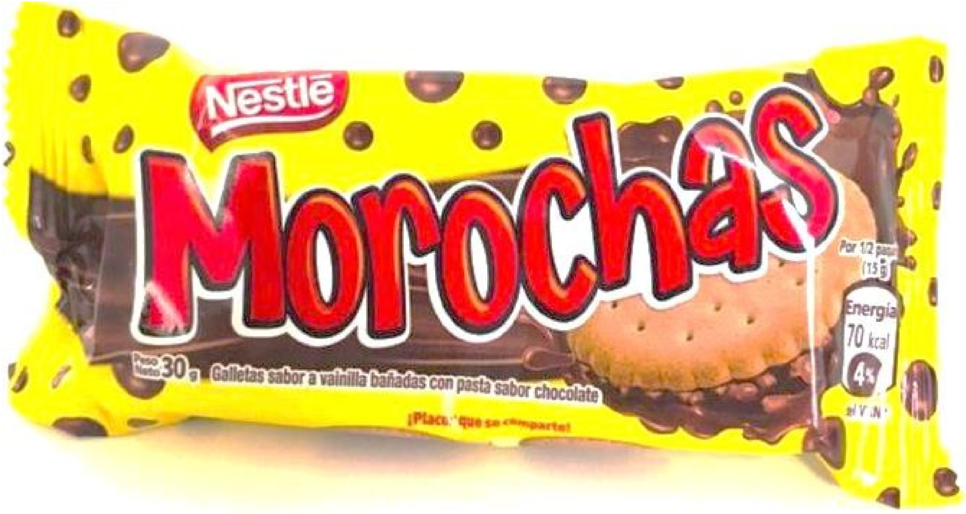

1 pack of sweet biscuits with chocolate filling or coating

3. CRACKERS

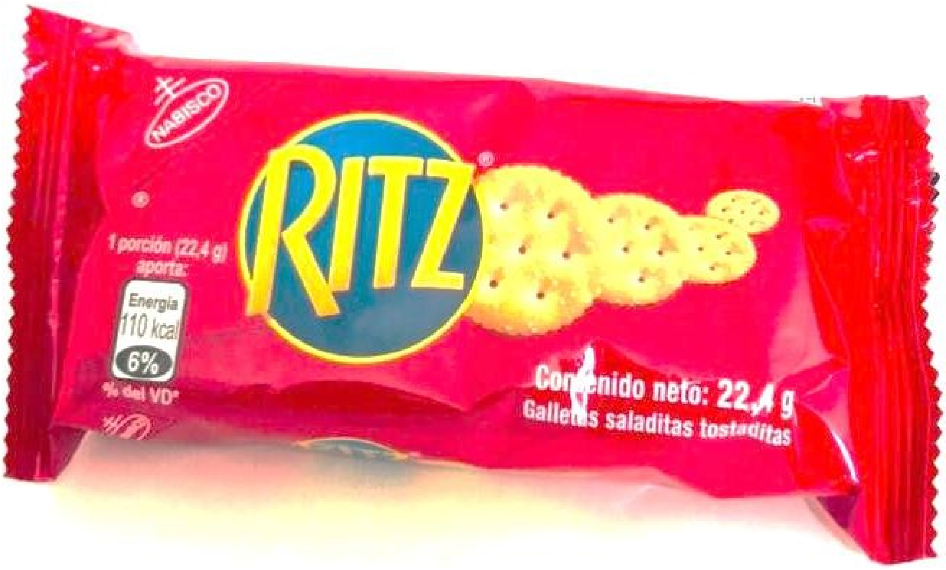

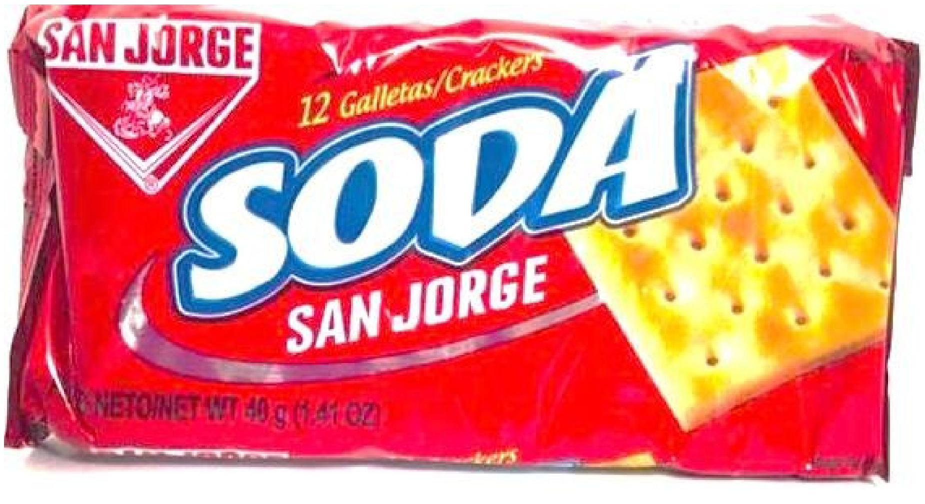

1 pack of savoury crackers

4. WHOLE GRAIN COOKIES

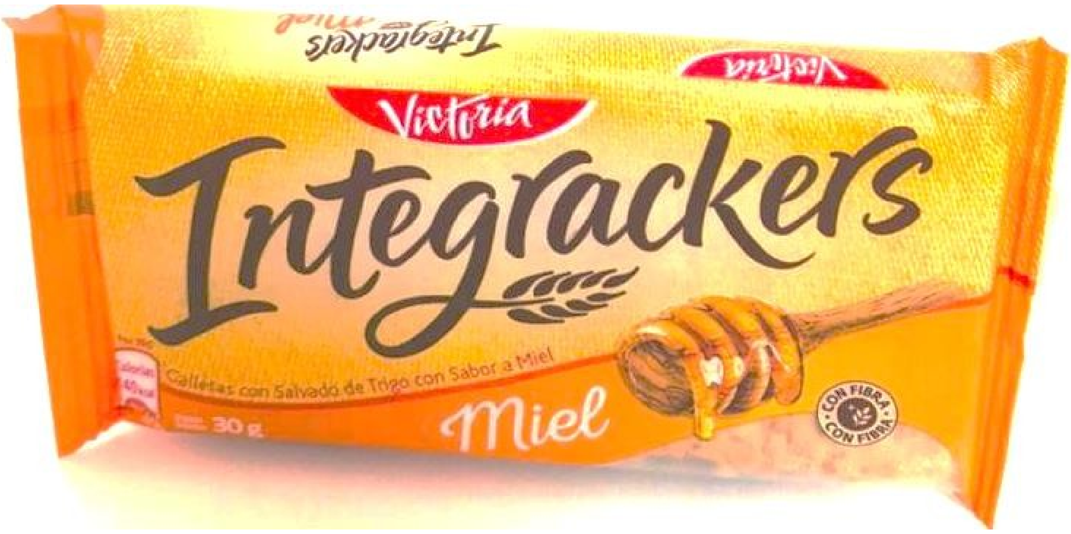

1 pack of whole grain cookies

5. ICE CREAM

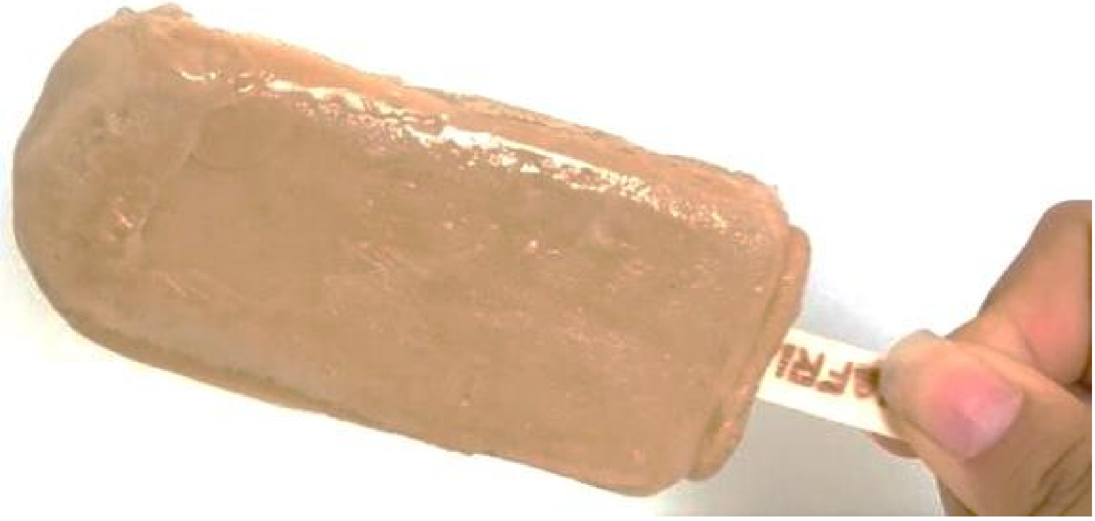

1 ice lolly or 1 scoop of ice cream

6. CHOCOLATES

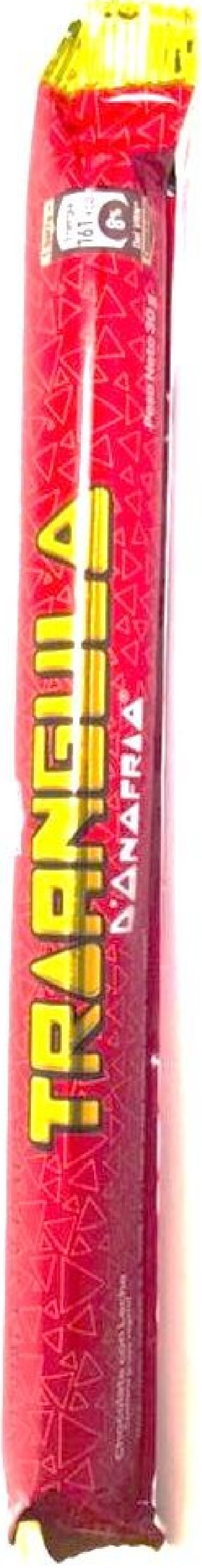

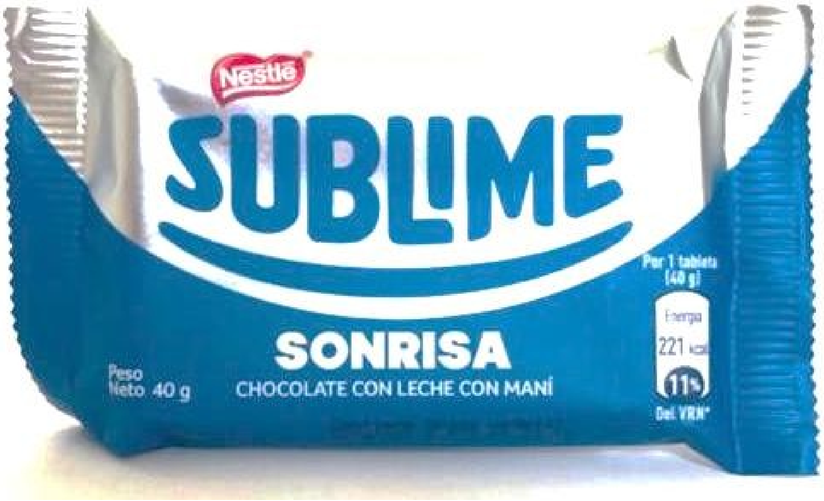

1 chocolate bar

7. CANDIES, TOFFEES AND GUMMIES

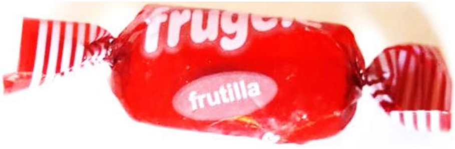

1 piece of candy

8. LOLLIPOPS

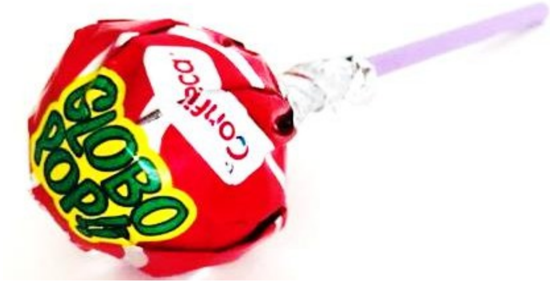

1 lollipop

9. YOGURT WITH CEREAL

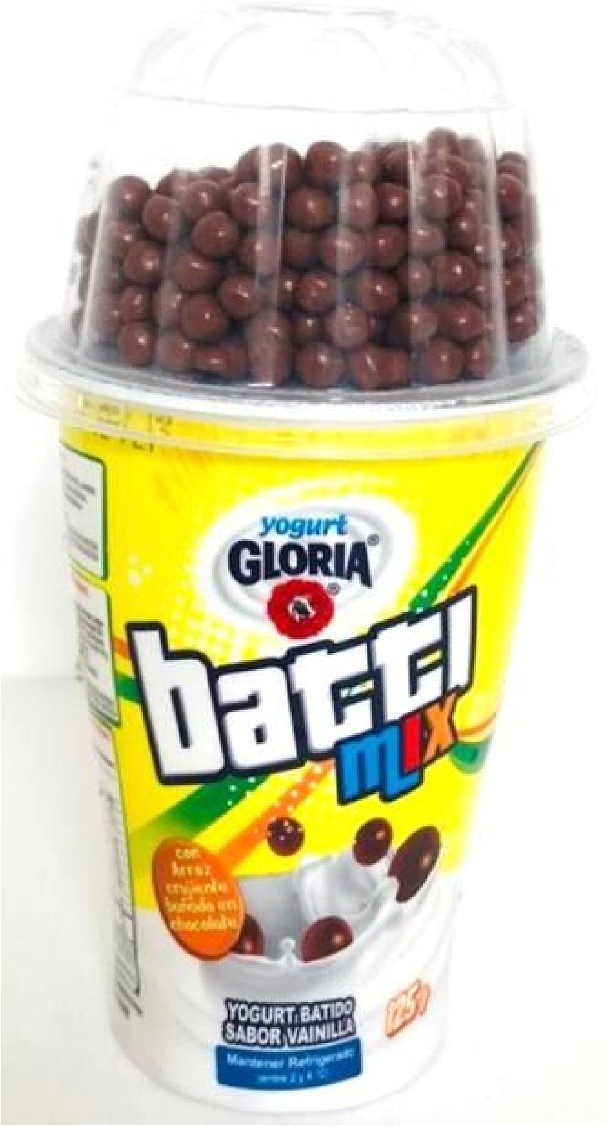

1 cup of yogurt with cereal

10. JELLY

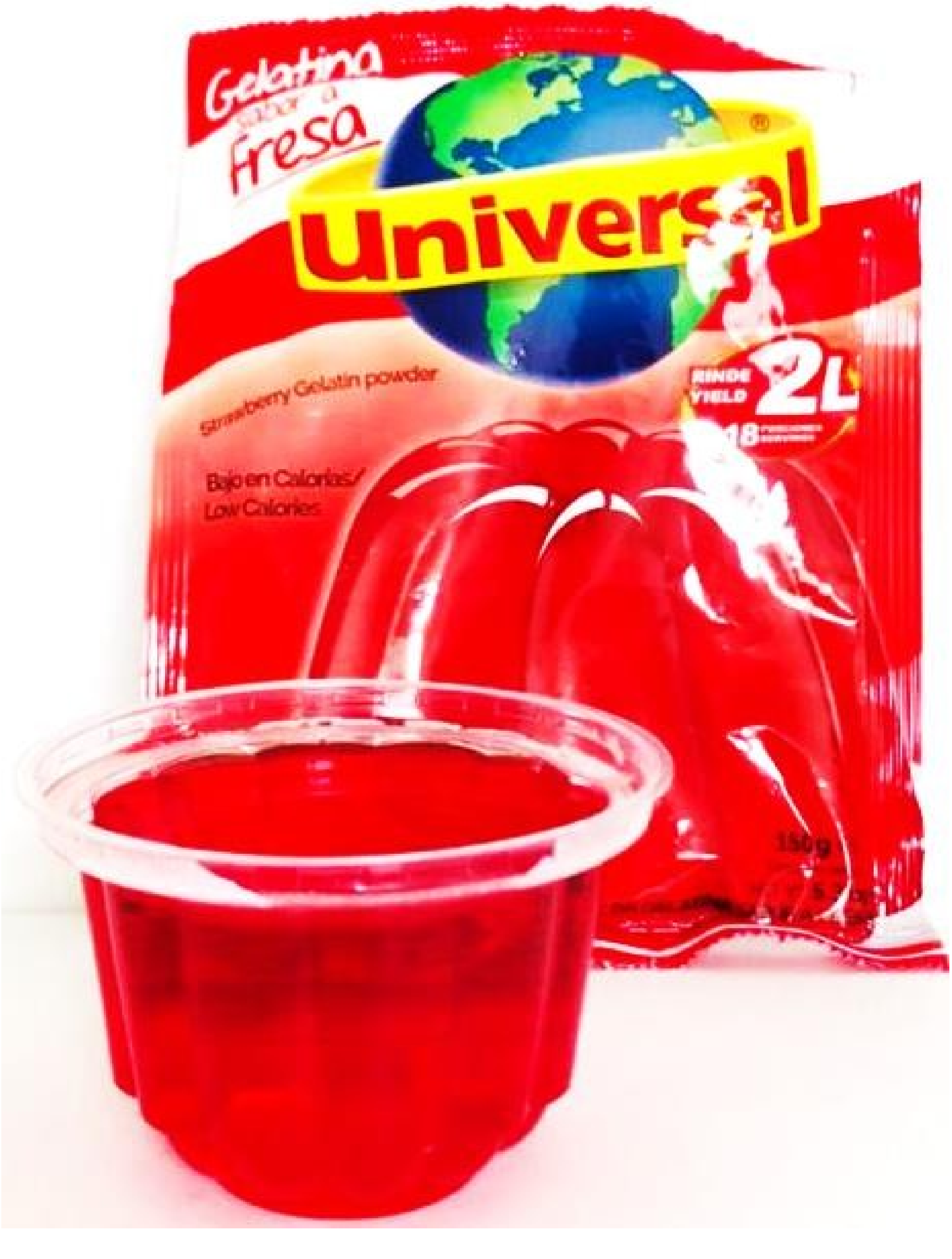

1 small cup of jelly

11. BREAKFAST CEREAL NO ADDED SUGAR

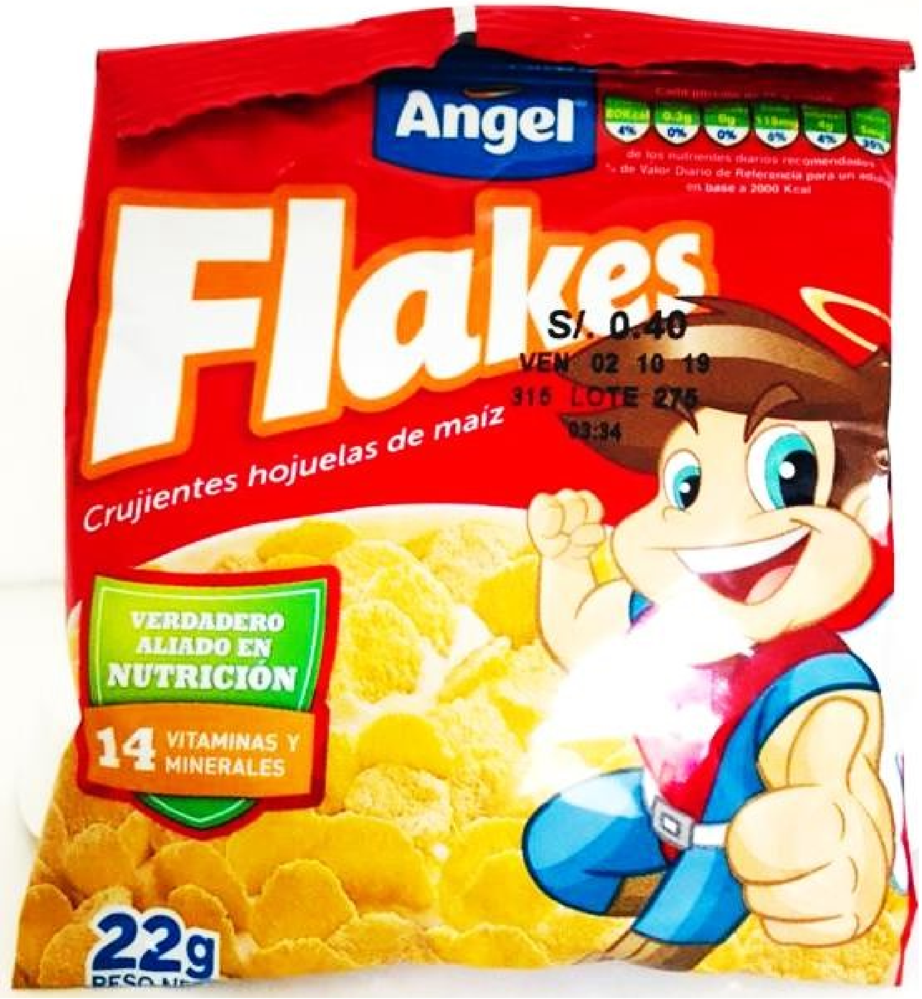

1 small bag of breakfast cereal (no added sugar)

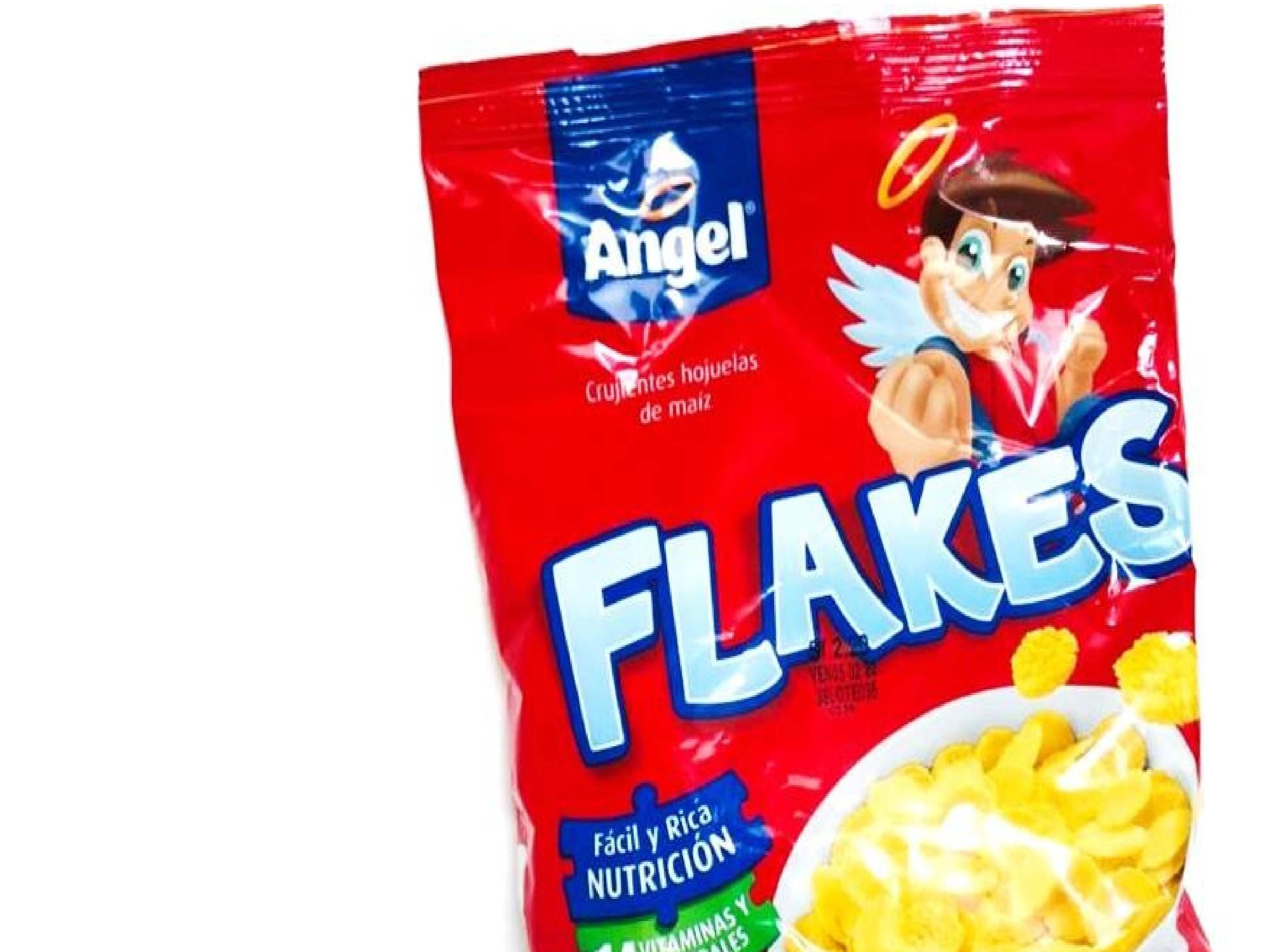

1 handful of breakfast cereal (no added sugar)

12. SWEETENED BREAKFAST CEREAL

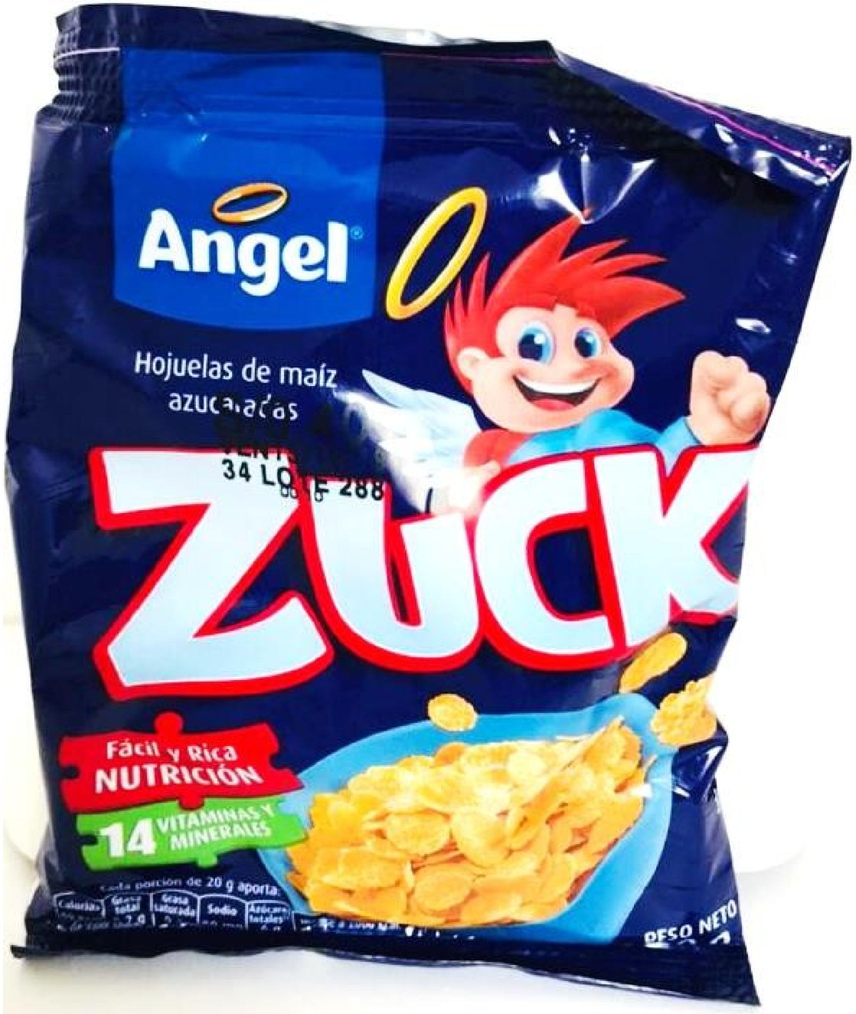

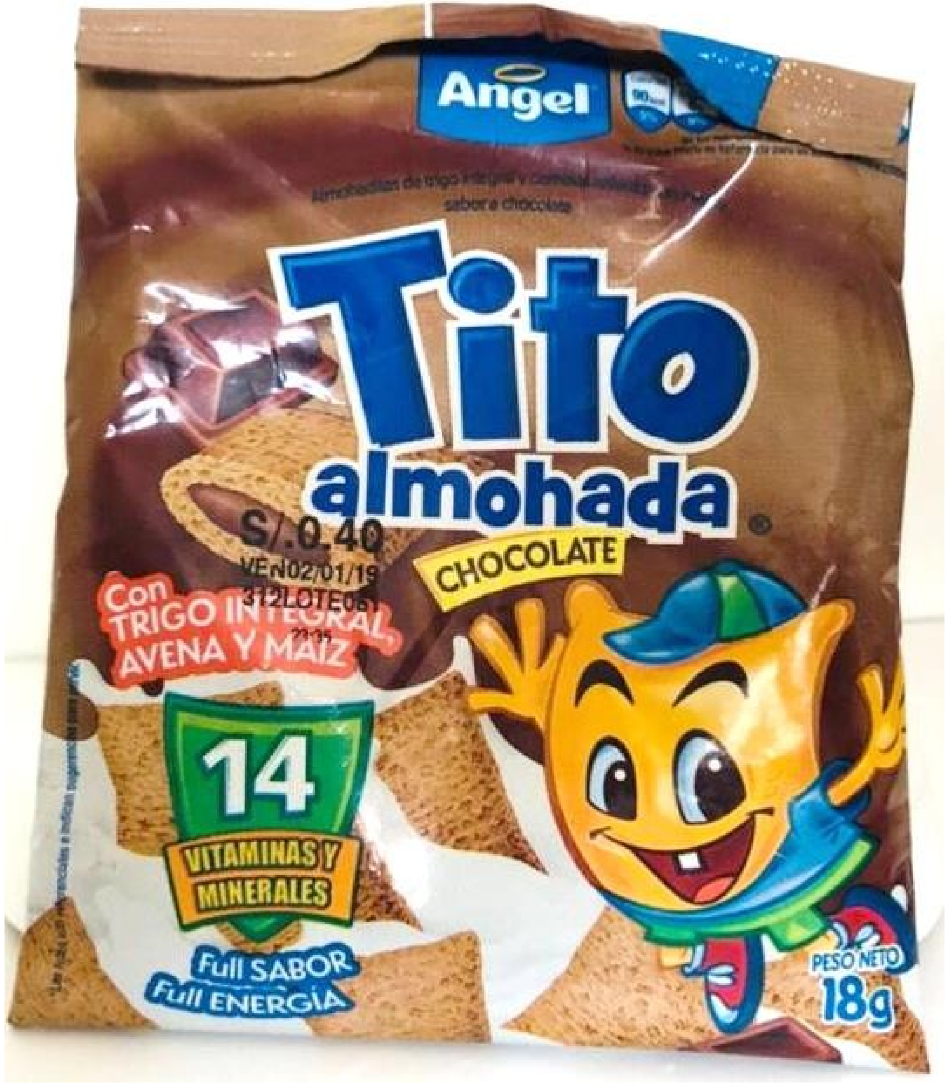

1 small bag of sweetened breakfast cereal

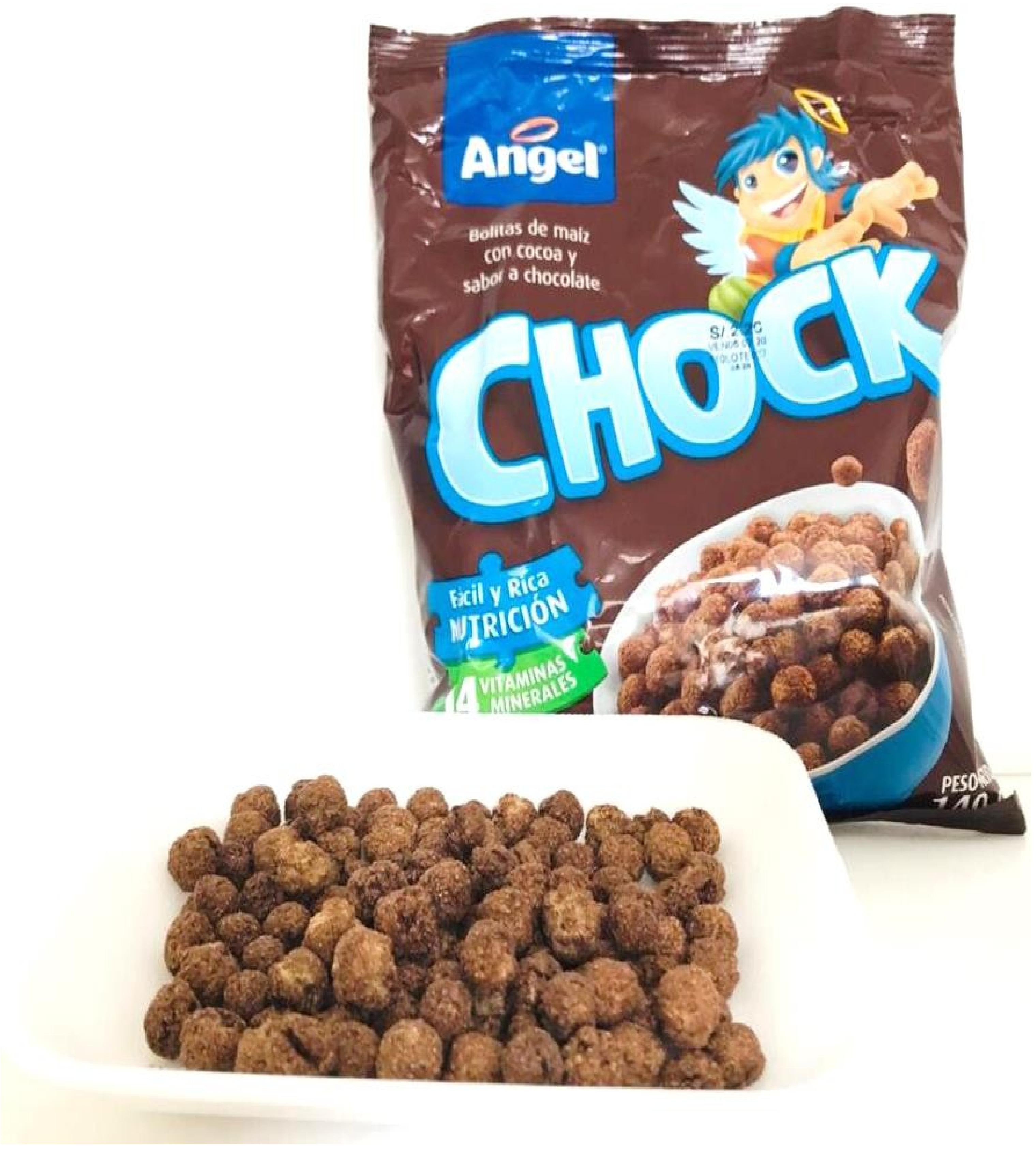

1 handful of sweetened breakfast cereal

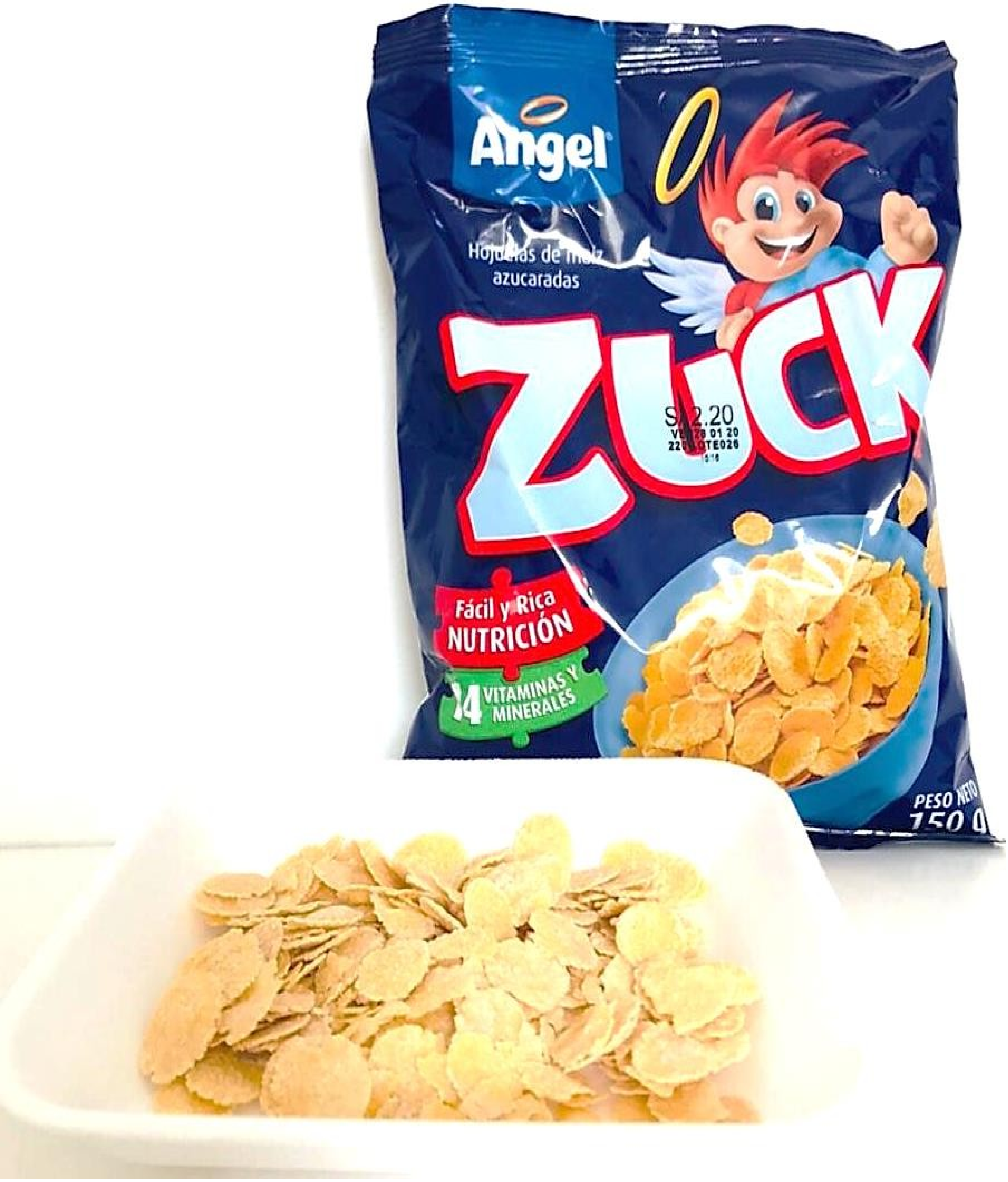

1 handful of sweetened breakfast cereal

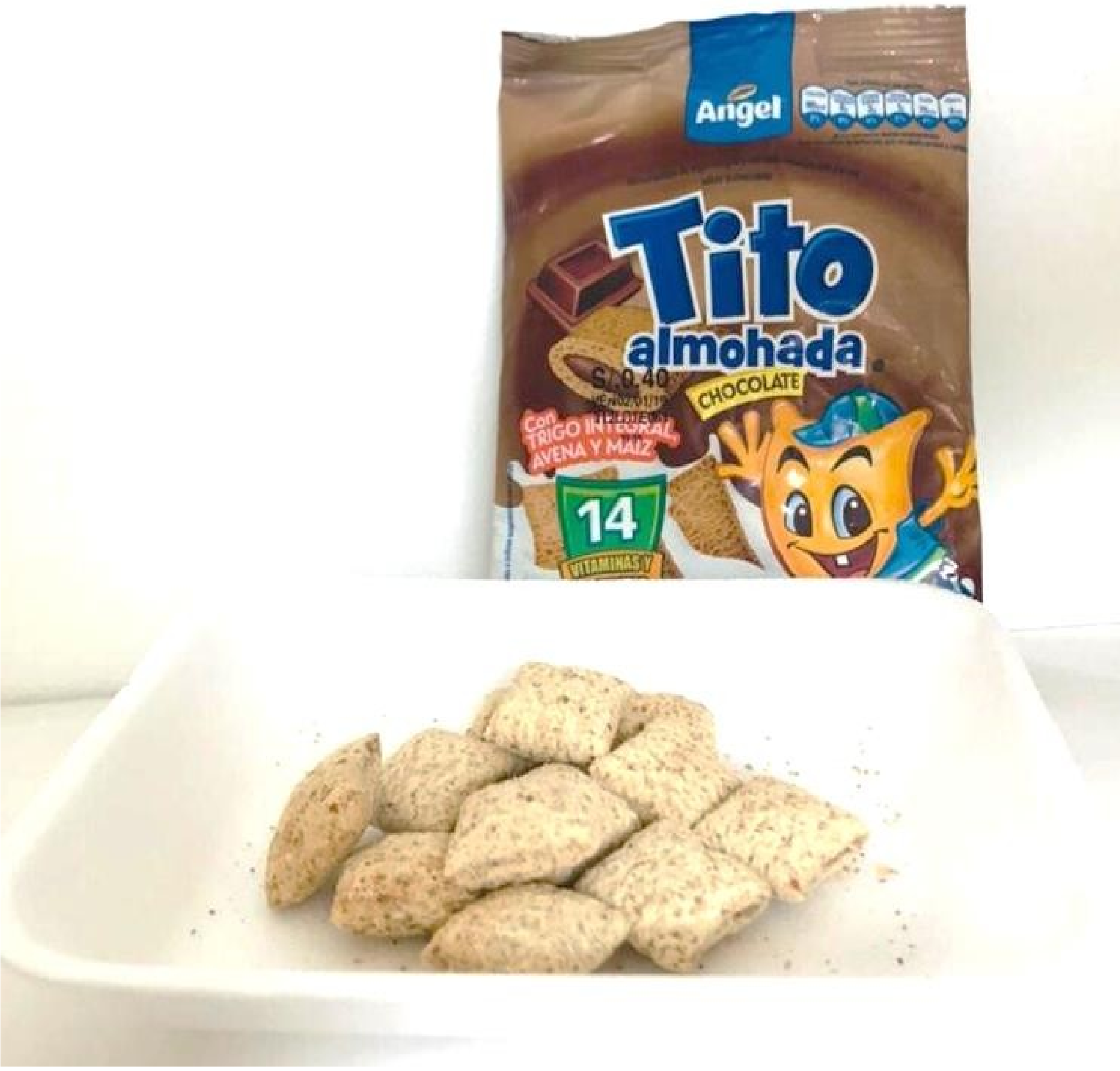

1 handful of sweetened breakfast cereal

13. CEREAL BARS

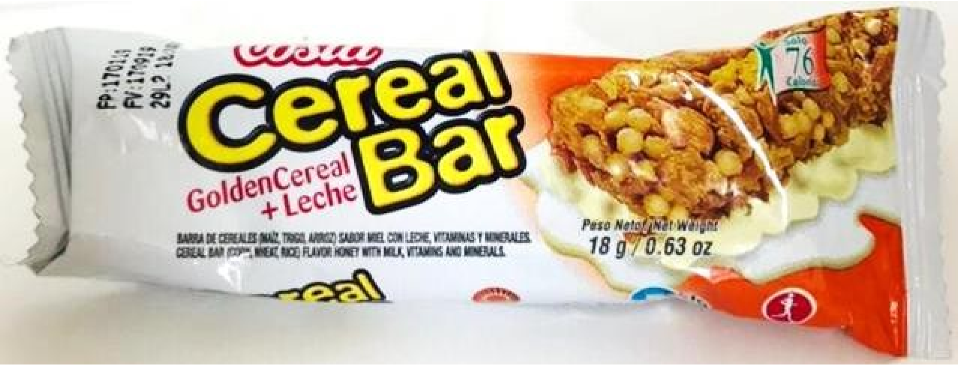

1 cereal bar

14. SALTY SNACKS

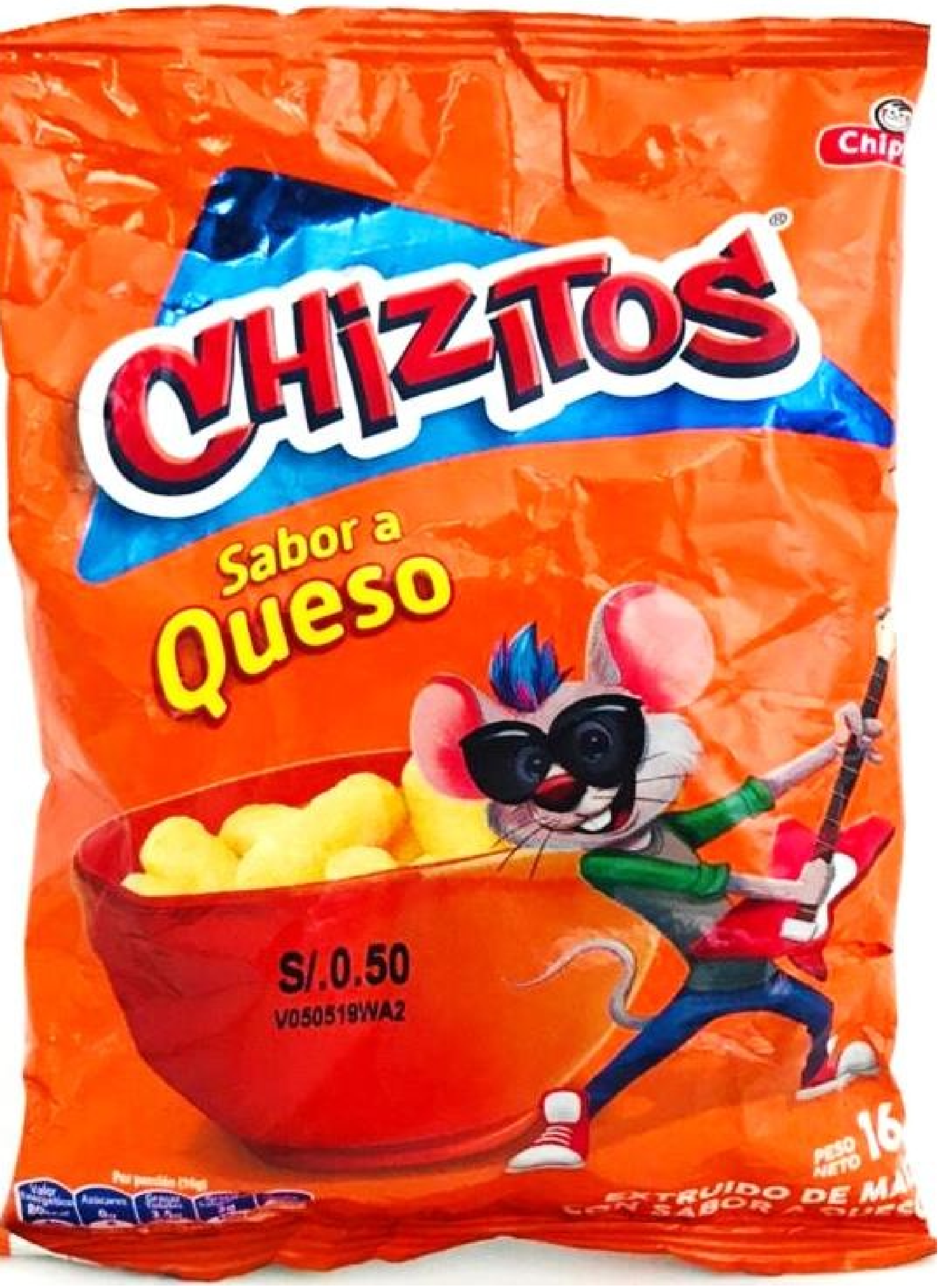

1 small bag of salty snacks

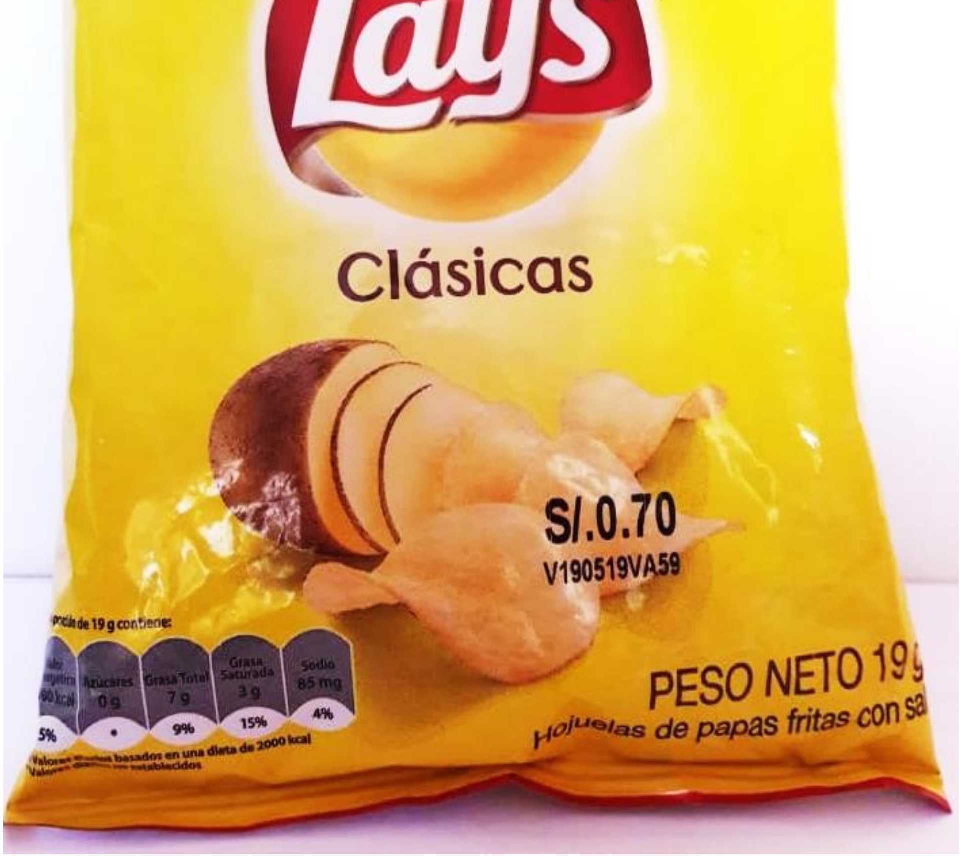

1 1 small bag of salty snacks

15. HOT DOG, PROCESSED HAM

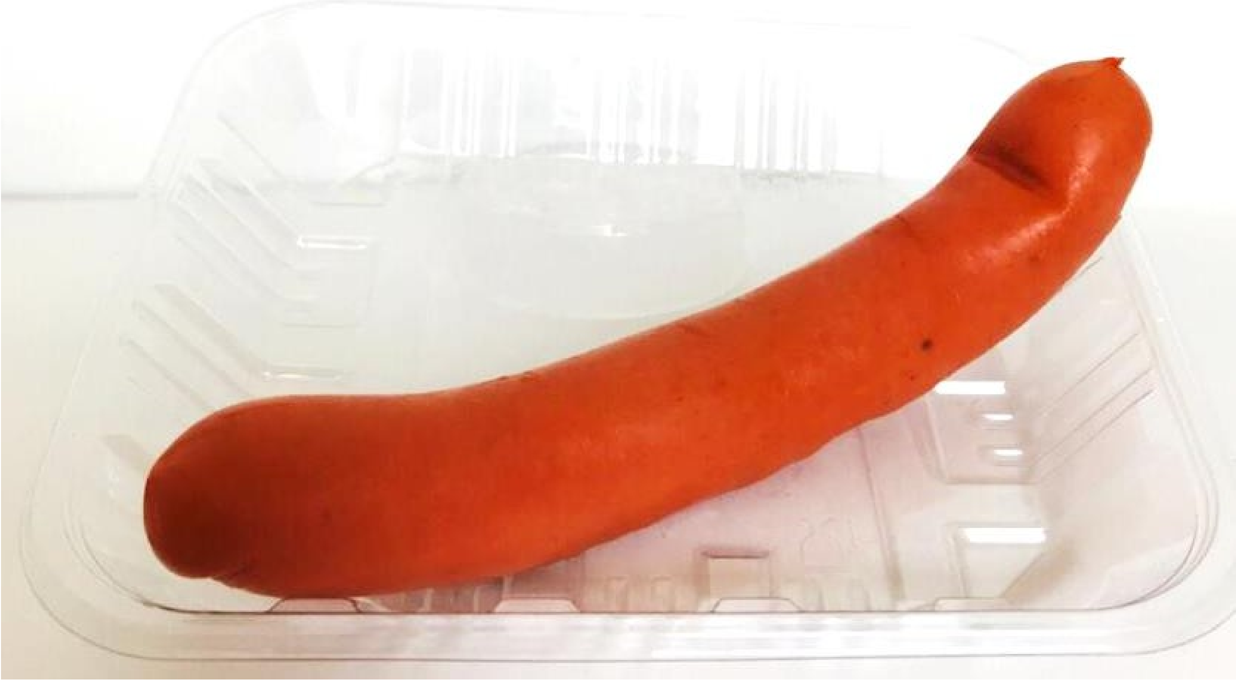

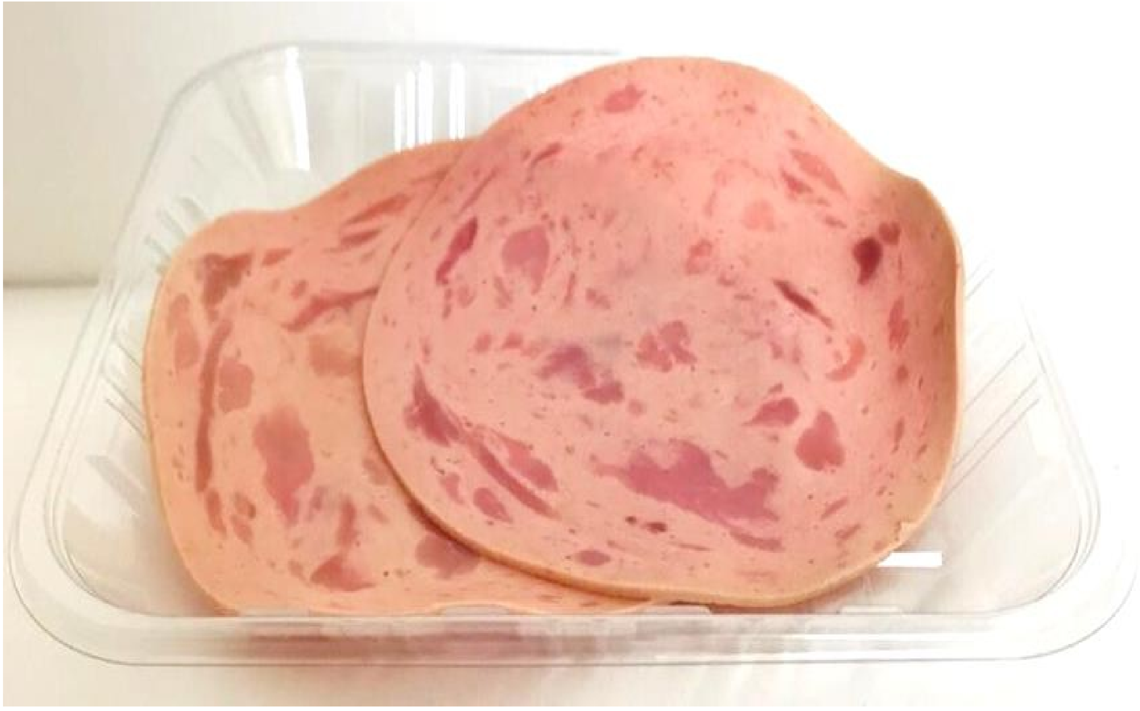

1 hot dog sausage or 2 slices of bologna

16. INSTANT SOUP

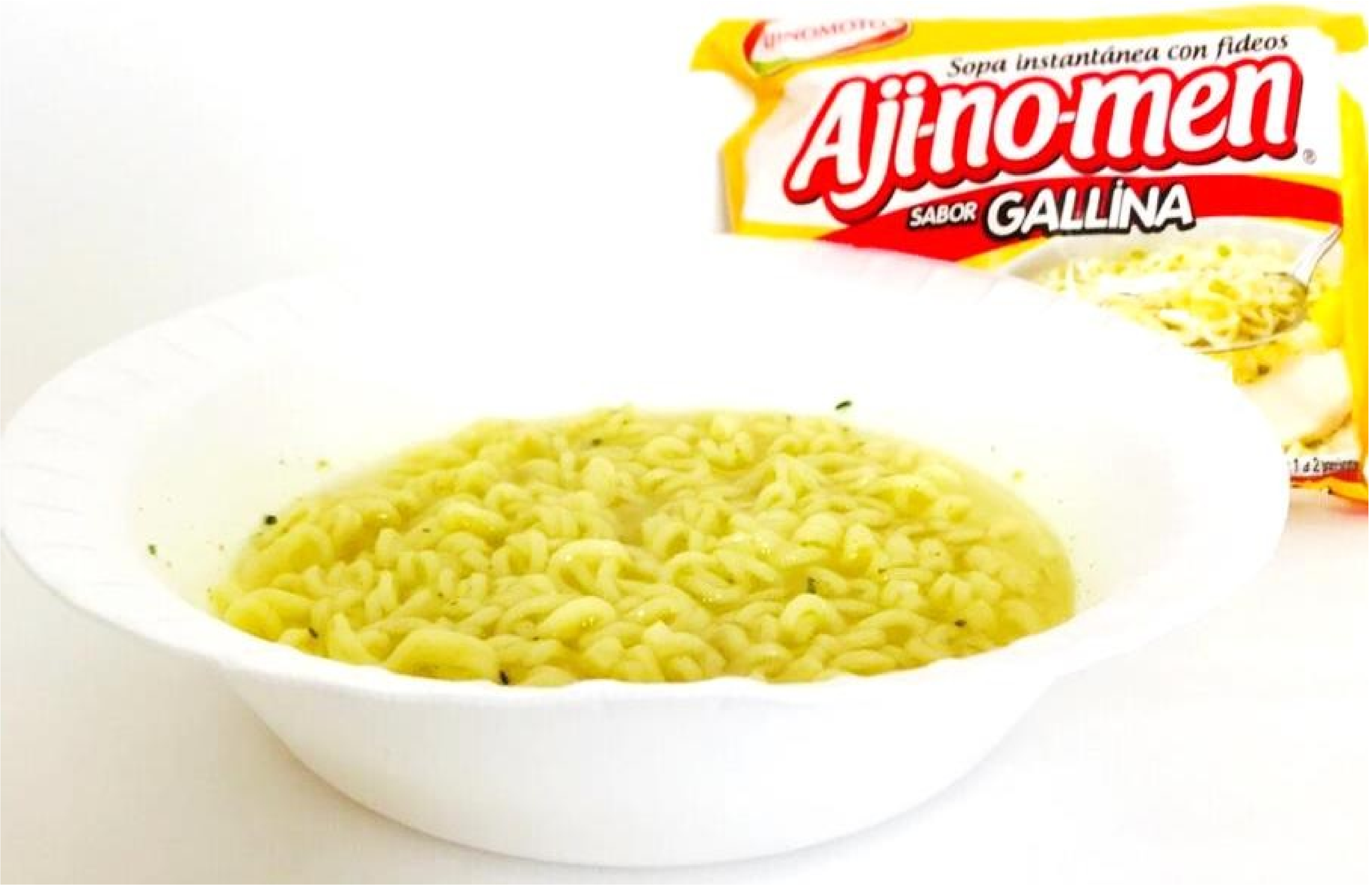

1 bowl of instant soup

17. CHICKEN NUGGETS

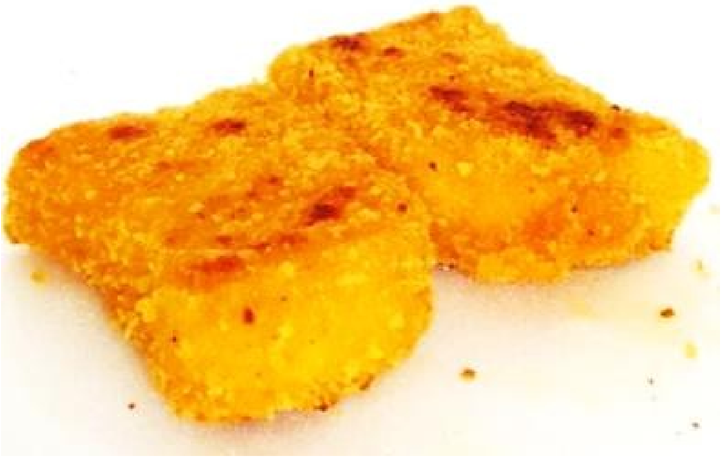

2 chicken nuggets

Supplementary Table S1. Sociodemographic characteristics of mothers and children who did and did not partipcate in the follow-up survey in 2021

Characteristics	Participated in 2019 & 2021 (n=1032)		Only participated in 2019 (n=251)		p-value**
	n or mean	% or SD	n or mean	% or SD	
**Mother characteristics**					
**Mother age***	36.29	7.2	35.41	7.1	0.12
**Mother education**					
**Primary (0-6 years)**	16	1.6	20	8.0	<0.001
**Secondary (7-11 years)**	310	30.0	97	38.6	
**Higher education (≥12 years)**	706	68.4	134	53.4	
**Marital status**					
**Married**	329	31.9	62	24.7	0.14
**Cohabiting**	482	46.7	127	50.6	
**Single**	163	15.8	48	19.1	
**Separated/Divorced/**	55	5.3	12	4.8	
**Widowed**	3	0.3	2	0.80	
**Socioeconomic level**					
**B**	511	49.5	108	43.0	0.06
**D**	521	50.5	143	57.0	
**Mother main occupation**					
**Working**	277	26.8	61	24.3	0.41
**Homemaker**	755	73.2	190	75.7	
**Number of children***	2.19	1.1	2.29	1.3	0.21
**Children characteristics**					
**Age of child at baseline***	4	0.8	3.9	0.8	0.53
**Sex of child**					
**Female**	476	46.1	115	45.8	0.93
**Male**	556	53.9	136	54.2	

*Mean (SD)

**T-test p-values for maternal age, number of children and age of child at baseline; chi-square p-values for mother education, marital status, socioeconomic level, mother main occupation and sex of child.

Supplementary Table S2. Spearman correlations between the Food Frequency Questionnaire and 24-hour dietary recalls for HFSS package products intake and nutrient estimates (n=118)

	Volume (g/ml)		Calories		Sodium (mg)		Sugar (g)		Saturated Fat (g)	
**Description**	**r**	**p-value**	**r**	**p-value**	**r**	**p-value**	**r**	**p-value**	**r**	**p-value**
Total food and beverages	0.16	0.09	0.35	<0.01	0.37	<0.01	0.30	<0.01	0.32	<0.01
Total beverages	0.15	0.10	0.41	<0.01	0.39	<0.01	0.37	<0.01	0.34	<0.01
**Water**	0.05	0.56	N.D.	N.D.	0.05	0.59	N.D.	N.D.	N.D.	N.D.
**Nectars and Flavored fruit drinks**	0.04	0.68	0.05	0.60	0.04	0.70	0.06	0.50	N.D.	N.D.
**Carbonated and sports drinks**	0.52	<0.01	0.34	<0.01	0.48	<0.01	0.34	<0.01	N.D.	N.D.
**Milk**	0.07	0.43	0.06	0.50	0.07	0.46	0.07	0.48	0.06	0.55
**Dairy drinks**	0.22	0.02	0.19	0.04	0.17	0.06	0.24	0.01	0.20	0.03
**Formulas and supplements**	0.43	<0.01	0.43	<0.01	0.43	<0.01	0.43	<0.01	0.43	<0.01
Total food	0.23	0.01	0.22	0.01	0.38	<0.01	0.05	0.63	0.19	0.04
**Salty crackers**	0.25	0.01	0.25	0.01	0.25	0.01	0.25	0.01	0.25	0.01
**Sweet crackers**	0.19	0.04	0.19	0.04	0.18	0.05	0.20	0.03	0.19	0.04
**Sweet pastries and bakery products**	-0.02	0.85	-0.02	0.85	-0.02	0.84	-0.02	0.84	-0.02	0.85
**Breakfast cereals and bars**	0.05	0.57	0.06	0.55	0.07	0.48	0.07	0.49	-0.09	0.35
**Candy and chewables**	0.23	0.01	0.23	0.01	0.23	0.01	0.24	0.01	0.18	0.05
**Desserts**	0.05	0.61	-0.02	0.80	0.05	0.61	-0.01	0.92	0.09	0.33
**Chocolate candies**	-0.03	0.73	-0.03	0.73	-0.04	0.70	-0.03	0.75	-0.04	0.71
**Soup**	-0.04	0.64	-0.04	0.64	-0.04	0.64	-0.04	0.64	-0.04	0.64

**Processed meats**	0.25	0.01	0.23	0.01	0.25	0.01	0.26	<0.01	0.23	0.01
**Dairy desserts**	0.13	0.15	0.14	0.13	0.15	0.12	0.14	0.13	0.15	0.12
**Snacks**	0.36	<0.01	0.36	<0.01	0.36	<0.01	0.39	<0.01	0.37	<0.01

Note. Missing cell values indicate zero values (e.g., calories for water). N.D.: nutrient not declared in the nutrition facts panel and therefore not estimated. For water, energy content is 0 kcal and does not represent an undeclared nutrient value.

Supplementary Table S3. Adjusted Spearman correlations between the Food Frequency Questionnaire and 24-hour dietary recalls for HFSS package products intake and nutrient estimates (n=118)

	Volume (g/ml)		Calories		Sodium (mg)		Sugar (g)		Saturated Fat (g)	
**Description**	**r**	**p-value**	**r**	**p-value**	**r**	**p-value**	**r**	**p-value**	**r**	**p-value**
Total food and beverages	0.19	0.05	0.33	<0.01	0.36	<0.01	0.29	<0.01	0.30	<0.01
Total beverages	0.18	0.05	0.41	<0.01	0.38	<0.01	0.38	<0.01	0.33	<0.01
**Water**	0.07	0.48	N.D.	N.D.	0.06	0.52	N.D.	N.D.	N.D.	N.D.
**Nectars and Flavored fruit drinks**	0.05	0.59	0.06	0.54	0.05	0.60	0.08	0.39	N.D.	N.D.
**Carbonated and sports drinks**	0.53	<0.01	0.35	<0.01	0.48	<0.01	0.35	<0.01	N.D.	N.D.
**Milk**	0.08	0.40	0.07	0.47	0.07	0.43	0.07	0.45	0.06	0.53
**Dairy drinks**	0.21	0.02	0.18	0.05	0.16	0.09	0.23	0.01	0.18	0.06
**Formulas and supplement**	0.43	<0.01	0.43	<0.01	0.43	<0.01	0.43	<0.01	0.43	<0.01
Total food	0.23	0.01	0.22	0.02	0.38	<0.01	0.04	0.68	0.19	0.04
**Salty crackers**	0.24	0.01	0.24	0.01	0.24	0.01	0.24	0.01	0.24	0.01
**Sweet crackers**	0.18	0.05	0.18	0.05	0.17	0.07	0.18	0.05	0.17	0.07
**Sweet pastries and bakery products**	-0.07	0.46	-0.07	0.46	-0.07	0.45	-0.07	0.45	-0.07	0.46
**Breakfast cereals and bars**	0.07	0.49	0.07	0.47	0.08	0.40	0.08	0.42	-0.08	0.41
**Candy and chewables**	0.23	0.01	0.23	0.01	0.23	0.01	0.24	0.01	0.18	0.06
**Desserts**	0.04	0.70	-0.04	0.69	0.04	0.69	-0.03	0.78	0.09	0.34
**Chocolate candies**	-0.04	0.69	-0.04	0.68	-0.04	0.65	-0.03	0.72	-0.04	0.65

**Soup**	-0.02	0.83	-0.02	0.83	-0.02	0.83	-0.02	0.83	-0.02	0.83
**Processed meats**	0.26	0.01	0.24	0.01	0.26	0.01	0.27	<0.01	0.24	0.01
**Dairy desserts**	0.13	0.15	0.14	0.13	0.15	0.12	0.14	0.13	0.15	0.12
**Snacks**	0.35	<0.01	0.35	<0.01	0.35	<0.01	0.39	<0.01	0.36	<0.01

Note: Adjusted for socioeconomic level (SEL), age, and sex. N.D.: nutrient not declared in the nutrition facts panel and therefore not estimated. For water, energy content is 0 kcal and does not represent an undeclared nutrient value.

Supplementary Table S4. Per consumer daily intake of HFSS packaged foods (grams) and beverages (milliliters), stratified by socioeconomic level in 2019 and 2021

		2019			2021			Difference between 2019 and 2021		
**Beverage/ food category**	**Consumers (n)**	**B Median (Q1-Q3)**	**D Median (Q1-Q3)**	**B vs D p-value***	**B Median (Q1-Q3)**	**D Median (Q1-Q3)**	**B vs D p-value***	**B Median difference†**	**D Median difference†**	**B vs D p-value***
**Total beverages**	1030	1481.3 (1015.9–2059.3)	1392.3 (971.2–1938.7)	0.123	1413.6 (1041.0–1984.6)	1440.3 (999.5–2014.7)	0.491	-67.7	48.0	0.113
**Water**	978	718.2 (450.0–1211.5)	718.2 (450.0–1125.0)	1.000	900.0 (536.5–1350.0)	900.0 (504.0–1350.0)	1.000	181.8	181.8	1.000
**Nectars and flavored fruit drinks**	475	32.9 (14.1–79.3)	32.9 (14.1–73.4)	1.000	32.9 (14.1–65.8)	31.5 (13.5–64.4)	0.819	0.0	-1.4	0.792
**Carbonated and sports drinks**	572	31.5 (13.5–57.0)	22.5 (13.5–51.9)	0.002	30.4 (13.5–63.0)	27.0 (13.5–63.0)	0.009	-1.1	4.5	0.107
**Teas**	792	94.5 (33.8–225.0)	112.5 (54.0–225.0)	0.177	126.0 (63.0–225.0)	94.5 (63.0–225.0)	0.555	31.5	-18.0	0.009
**Milk**	420	450.0 (94.5–675.0)	225.0 (63.0–450.0)	<0.001	225.0 (94.5–450.0)	157.5 (63.0–450.0)	<0.001	-225.0	-67.5	<0.001
**Dairy drinks**	841	170.8 (75.6–470.0)	151.2 (63.9–360.0)	0.268	133.0 (63.0–283.5)	113.4 (50.4–236.3)	0.093	-37.8	-37.8	1.000
**Formulas and**	50	337.5 (225.0–500.0)	202.5 (94.5–450.0)	0.069	225.0 (101.3–450.0)	157.5 (31.5–225.0)	0.065	-112.5	-45.0	0.402

supplements										
**Total food**	1026	56.5 (27.6–92.1)	56.9 (30.5–92.6)	0.921	46.2 (24.7–73.9)	42.0 (21.8–77.2)	0.460	-10.4	-14.9	0.238
**Salty crackers**	488	4.4 (1.9–8.8)	4.4 (2.2–8.8)	1.000	4.4 (1.9–8.8)	4.4 (1.9–8.8)	1.000	0.0	0.0	1.000
**Sweet crackers**	729	5.4 (3.2–11.0)	4.9 (2.5–9.8)	0.296	6.7 (3.2–11.6)	4.9 (3.0–9.8)	0.149	1.3	0.0	0.330
**Sweet pastries and bakery products**	276	5.1 (2.6–8.0)	4.0 (1.7–8.0)	0.141	5.1 (3.4–8.0)	8.0 (3.4–12.0)	0.150	0.0	4.0	<0.001
**Breakfast cereals and bars**	552	5.6 (1.8–8.4)	5.6 (2.8–11.2)	1.000	5.6 (1.8–11.2)	5.2 (2.4–8.4)	0.710	0.0	-0.4	0.651
**Candy and chewables**	475	2.0 (0.9–4.1)	1.8 (0.9–3.3)	0.456	1.8 (0.9–3.5)	1.6 (0.8–3.5)	0.361	-0.2	-0.2	0.918
**Desserts**	187	20.7 (6.9–48.3)	32.2 (10.4–64.4)	0.069	13.8 (6.9–31.1)	16.1 (6.9–32.2)	0.059	-6.9	-16.1	0.144
**Chocolate candies**	443	2.2 (0.9–4.3)	1.9 (0.9–4.3)	0.147	2.2 (0.9–4.3)	2.2 (0.9–4.3)	0.359	0.0	0.3	0.248
**Instant soups**	72	24.0 (12.0–56.0)	24.0 (12.0–56.0)	1.000	24.0 (24.0–56.0)	48.0 (12.0–56.0)	0.231	0.0	24.0	0.049
**Processed meats**	681	6.6 (2.8–13.2)	6.7 (3.7–13.2)	0.973	8.8 (3.8–15.0)	7.5 (3.8–13.2)	0.461	2.2	0.9	0.343

**Dairy desserts**	543	19.0 (6.0–38.1)	16.3 (8.2–30.6)	0.113	11.8 (4.8–20.2)	8.2 (4.1–21.4)	0.006	-7.3	-8.2	0.654
**Snacks**	480	1.2 (0.5–2.5)	1.2 (0.5–2.5)	1.000	1.6 (1.1–2.5)	1.6 (0.5–2.5)	1.000	0.3	0.4	1.000

Note: Values include participants who consumed the corresponding category in both 2019 and 2021; therefore, the number of participants varies across categories. *P-values were obtained from quantile regression models with standard errors clustered by child identifier. For the 2019 and 2021 columns, the p-values compare median intake between socioeconomic groups B and D within each wave. For the difference between 2019 and 2021, the final p-value tests whether the between-wave difference in median intake differs between socioeconomic groups B and D. †Medians and Q1–Q3 were estimated separately for each socioeconomic group and survey wave using quantile regression. Median difference represents the difference between the wave-specific medians.
